# Hybrid Epigenomes Reveal Extensive Local Genetic Changes to Chromatin Accessibility Contribute to Divergence in Embryonic Gene Expression Between Species

**DOI:** 10.1093/molbev/msad222

**Published:** 2023-10-12

**Authors:** Hannah R Devens, Phillip L Davidson, Maria Byrne, Gregory A Wray

**Affiliations:** Department of Biology, Duke University, Durham, NC, USA; Department of Biology, Duke University, Durham, NC, USA; School of Medical Science, The University of Sydney, Sydney, New South Wales, Australia; School of Life and Environmental Science, The University of Sydney, Sydney, New South Wales, Australia; Department of Biology, Duke University, Durham, NC, USA; Center for Genomic and Computational Biology, Duke University, Durham, NC, USA

**Keywords:** evolution, development, gene regulation, sea urchins, life history, ATAC-seq, hybrid

## Abstract

Chromatin accessibility plays an important role in shaping gene expression, yet little is known about the genetic and molecular mechanisms that influence the evolution of chromatin configuration. Both local (*cis*) and distant (*trans*) genetic influences can in principle influence chromatin accessibility and are based on distinct molecular mechanisms. We, therefore, sought to characterize the role that each of these plays in altering chromatin accessibility in 2 closely related sea urchin species. Using hybrids of *Heliocidaris erythrogramma* and *Heliocidaris tuberculata*, and adapting a statistical framework previously developed for the analysis of *cis* and *trans* influences on the transcriptome, we examined how these mechanisms shape the regulatory landscape at 3 important developmental stages, and compared our results to similar analyses of the transcriptome. We found extensive *cis*- and *trans*-based influences on evolutionary changes in chromatin, with *cis* effects generally larger in effect. Evolutionary changes in accessibility and gene expression are correlated, especially when expression has a local genetic basis. Maternal influences appear to have more of an effect on chromatin accessibility than on gene expression, persisting well past the maternal-to-zygotic transition. Chromatin accessibility near gene regulatory network genes appears to be distinctly regulated, with *trans* factors appearing to play an outsized role in the configuration of chromatin near these genes. Together, our results represent the first attempt to quantify *cis* and *trans* influences on evolutionary divergence in chromatin configuration in an outbred natural study system and suggest that chromatin regulation is more genetically complex than was previously appreciated.

## Introduction

Chromatin configuration plays a critical role in transcriptional regulation in eukaryotes by enabling the ability of regulatory elements to influence transcription (Kornberg and Lorch 1992; Giacoman-Lozano, et al. 2022). Over the past decade, genome-wide assays (Zaret 2005; Song and Crawford 2010; Buenrostro, et al. 2015a; Cusanovich, et al. 2018) have revealed that chromatin accessibility is both highly dynamic and highly context-dependent. Extensive changes in the complement of open chromatin regions (OCRs) take place during development (Cusanovich, et al. 2018; Reddington, et al. 2020), leading to fully differentiated cells that typically differ in the majority of OCRs (Yue, et al. 2014; Buenrostro, et al. 2015b). Further changes in the accessibility of individual OCRs take place across circadian cycles and in response to a wide range of physiological conditions and external stimuli. The sheer scale of this remodeling is enormous: of the >800,000 OCRs known in humans, for instance, the vast majority are only accessible under a few conditions or in a small number of cell types or developmental stages (Thurman, et al. 2012; Jiang, et al. 2022).

Despite its importance as a mechanism contributing to transcriptional regulation, the evolutionary significance of chromatin remodeling remains poorly understood. Several studies have compared the open chromatin landscape among closely related species, in which it is possible to identify orthologous noncoding regions of the genome with high confidence (Shibata et al. 2012; Pizzollo et al. 2018; Edsall et al. 2019; Lewis and Reed 2019; Swain-Lenz et al. 2019; Davidson et al. 2022; Yao et al. 2022). These studies demonstrate that most OCRs are conserved in position and degree of accessibility among closely related species (<10 million years diverged), while a smaller proportion are conserved over longer time frames (Yue et al. 2014; Gao et al. 2018; Davidson et al. 2022). Yet, these same studies still find that hundreds or even thousands of OCRs are differentially accessible (DA) in the same cell type or tissue under the same conditions, even among closely related species. Some studies report a statistical association between whether an OCR is DA and whether the nearest gene is differentially expressed (DE) between species (Pizzollo et al. 2018; Davidson et al. 2022). This finding suggests that evolutionary changes in chromatin configuration may contribute to divergence in gene expression. That said, the association is generally weak and does not provide information about individual OCRs and genes. More importantly, it remains unclear what molecular mechanisms underlie species differences in chromatin, to what extent these differences are heritable, and how often heritable differences influence gene expression.

**Fig. 1.**
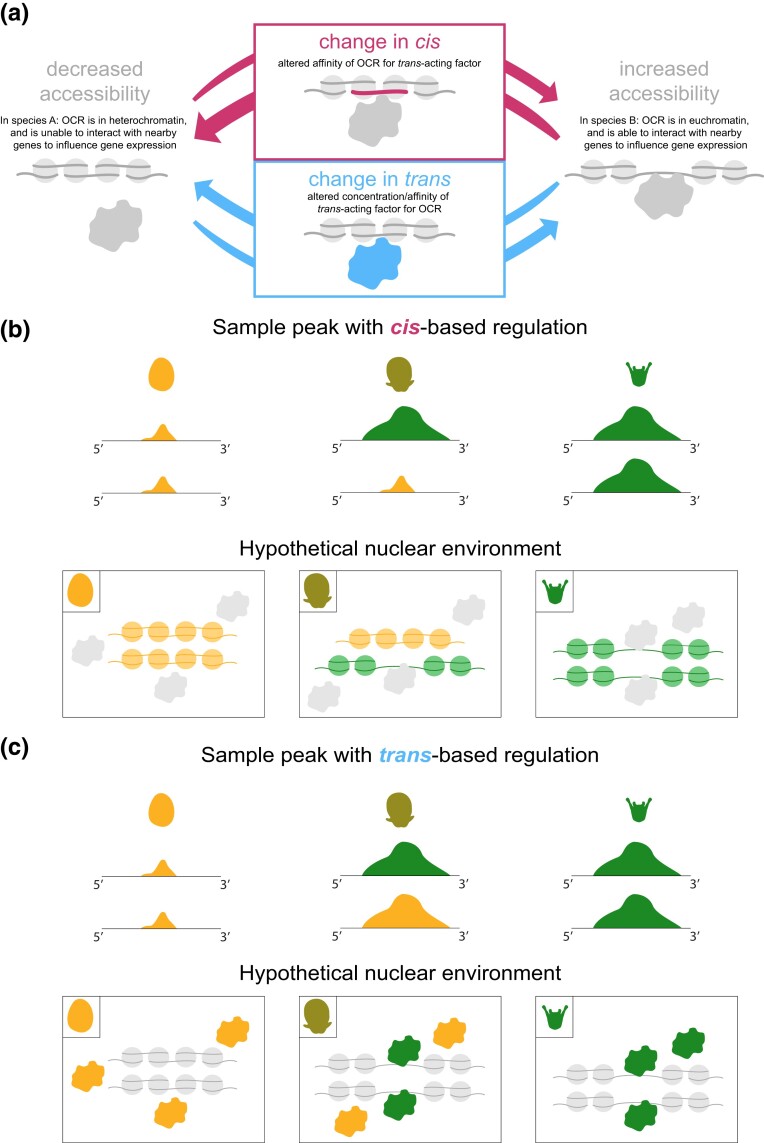
Conceptual overview of molecular mechanisms that could produce differences in chromatin accessibility between species. While several distinct molecular processes can modulate chromatin configuration, these can be grouped into 2 broad categories: those that are genetically based near the OCR of interest (*cis*) and those based elsewhere in the genome (*trans*). a) *Cis*-based changes (magenta here and in subsequent figures) are likely caused by a local mutation that alters the binding or interaction of an already-present protein (the alternative is a trans-generational nongenetic influence on chromatin that differs between species; such influences are likely to be uncommon based on their incidence within well studies species). Depending on whether the mutation raises or lowers binding affinity, and the protein's biochemical function, the consequence could be either an increase or decrease in accessibility at a specific genomic location (bidirectional arrows). *Trans*-based changes (light blue here and in subsequent figures) can be caused by a mutation that either alters the amino acid sequence or post-translational processing of a protein and thereby modifies its function, or by a change in the presence or concentration of a protein. Again, depending on the specific nature of these changes, chromatin accessibility at a specific location in the genome could either increase or decrease (bidirectional arrows). b) Model of *cis*-based change. In the *He* same-species cross (left panels), DNA is tightly wound around nucleosomes, and thus *trans*-acting factors (proteins) are unable to interact with it, resulting in very small peaks on both alleles. In the *Ht* same-species cross (right panels), DNA is not wrapped around nucleosomes, leaving it accessible to *trans*-acting factors, and thus generating 2 large peaks in the corresponding browser tracks. In hybrids (center), 1 allele from each parent is inherited, yielding 1 large peak and 1 small peak as the 2 alleles differ in their ability to interact with *trans*-acting factors. c) Model of *trans*-based change. In the *He* same-species cross (left panels), DNA is tightly wound around nucleosomes, and *trans*-acting factors are unable to interact with it, resulting in very small peaks on both alleles. In the *Ht* same-species cross (right panels), *trans*-acting factors (which differ from *He trans*-acting factors) are able to open up the chromatin and interact with it, thus generating 2 large peaks in the corresponding browser tracks. In the hybrid cross (center), *trans*-acting factors from each parent are present and able to interact with alleles inherited from either parent. Thus, the *Ht trans*-acting factors are able to open up the chromatin on both alleles, generating 2 equal-sized peaks in the browser tracks for the hybrid cross. Right, real examples of browser tracks correspond to distinct regulatory modes.

The mechanistic basis for differences in chromatin accessibility can be thought of as occurring in 2 different ways. One is through changes in *cis*: modifications to the nucleotide sequence of *cis*-regulatory elements themselves. The second is through changes in *trans:* alterations to the structure, localization, or expression of transcriptional regulators that interact with *cis*-regulatory elements (see Fig. 1). However, the relative contribution of these distinct molecular mechanisms to changes in chromatin accessibility differences remains unclear. Understanding the role that each of these mechanisms plays in changing chromatin accessibility is crucial to our understanding of how chromatin accessibility evolves because they represent 2 fundamentally different explanations for alterations to chromatin status: *trans* changes may be caused by either a zygotic or a maternal factor, and could be the result of a structural or an expression change; however, the majority of *cis* changes are likely due to local genetic changes (Fig. 1). The distinction may matter for natural selection, since *cis* changes are less likely to be pleiotropic, because their effects on traits are likely mediated only by one or a few genes. Thus, it is important to establish whether evolutionary differences in OCRs are heritable and whether they are consequential for local gene expression. If, instead, most are merely a reflection of genetic changes elsewhere in the genome or in the individual's parentage (in the case of maternal effects), or if they rarely influence local gene expression, evolutionary differences in OCRs likely contribute relatively little to trait evolution and adaptation.

In this study, we used interspecies hybrids of 2 sea urchin species (*Heliocidaris erythrogramma* and *Heliocidaris tuberculata*) to measure genetic contributions to divergence in chromatin configuration and the relationship of chromatin configuration to gene expression during embryonic development. We took advantage of a “natural experiment” in the evolution of gene expression driven by a recent life history switch from planktotrophy (feeding larvae) to lecithotrophy (nonfeeding larvae) within this genus (Raff and Byrne 2006; Wray 2022). This life history switch produced an unusually high concentration of recent evolutionary changes in gene expression (Israel et al. 2016) and chromatin configuration (Davidson et al. 2022) on the branch leading to lecithotrophy (*H. erythrogramma*). Positive selection is enriched in this branch, providing the ability to contrast neutral and adaptive changes in gene regulation (Israel et al. 2016; Davidson et al. 2022; Wray 2022). The evolution of lecithotrophy also involved extensive changes in maternal provisioning of metabolites and informational molecules (Hoegh-Guldberg and Emlet 1997; Byrne et al. 1999; Israel et al. 2016; Davidson et al. 2019). These recent, extensive changes in the molecular composition of eggs also allow us to test whether divergence in chromatin configuration is associated with changes in gene expression or is simply an indirect consequence of altered physiology with little relevance for the evolution of gene expression. Furthermore, the well-defined developmental gene regulatory network (GRN) of sea urchins (Davidson et al. 2002; Oliveri et al. 2008; Su et al. 2009; Peter and Davidson 2011; Rafiq et al. 2012) affords the opportunity to examine the architecture of *trans* effects in detail and to examine how natural selection operates on the transcriptional regulation of critical developmental genes.

We compared the open chromatin landscape during embryonic development in hybrids with those of same-species parental crosses, adapting a well-established statistical framework for the analysis of hybrid transcriptomes (Wittkopp et al. 2008; McManus et al. 2010; Pirinen et al. 2015) to the analysis of hybrid epigenomes. Taken together, our results emphasize differences in the regulation of the epigenome relative to the regulation of gene expression, support the idea that genetically based changes in chromatin contribute to the evolutionary divergence of gene expression, and highlight several distinct evolutionary properties of core promoters and OCRs near GRN genes.

## Results

### Reads From Hybrid Embryos Reflect Expected Biological Signals, Including Reproducing Parent-of-origin Patterns

**Fig. 2.**
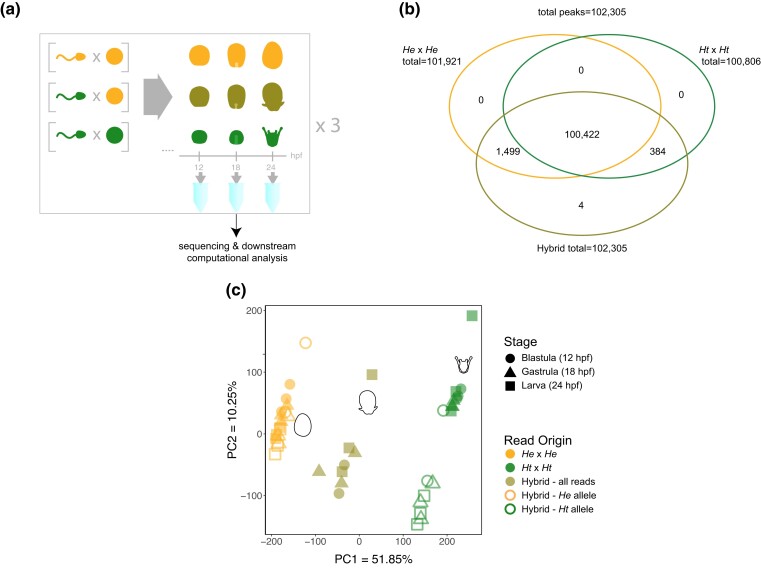
Chromatin configuration in parents and hybrids. a) Experimental design and workflow. Samples from 3 biological replicates of 3 genetic crosses (*He* × *He*, the maternal same-species cross; *Ht* × *Ht*, the paternal same-species cross; and *He* (female)×*Ht* (male), the hybrid cross) were collected at 3 timepoints (12, 18, 24 hpf: hours post fertilization). b) Venn diagram (not area-proportional) of peaks that are unique versus. shared among the same-species crosses and the hybrid cross. The reported peak count is the number of peaks following low-count removal. c) PCA of ATAC-seq results generated from counts table of reads in OCRs. Throughout this study, orange indicates *He* origin; green *Ht* origin; and olive hybrid origin.

We generated (Assay for Transposase-Accessible Chromatin (ATAC)-seq libraries from maternal *Heliocidaris erythrogramma* (*He*) × paternal *H. tuberculata* (*Ht*) hybrid embryos from 3 independent crosses at 3 stages of development (Fig. 2a) and assigned reads to parental genomes (see Methods). We refer to these crosses, respectively, as either “hybrids” or “same-species” throughout. The stages sampled match those in our earlier analysis of transcriptomes in the same hybrid cross (Wang et al. 2020) and are a subset of stages examined in comparative transcriptome and epigenome time courses for the 2 *Heliocidaris* species and an outgroup, *Lytechinus variegatus* (Israel et al. 2016; Davidson et al. 2022). We present data here from only one direction of interspecies hybrids because the reverse cross (maternal *Ht* × paternal *He*) arrests during gastrulation (Raff, et al. 1999). Before quality filtering, an average of 90.3% of reads from hybrids could be confidently assigned to a parental genome using this workflow (see Methods, supplementary Table S3, Supplementary Material online). This is a marked improvement over the approach we previously used in the transcriptome, which was able to map only 81.9% of reads to a parental genome. On average, 57.1% of our aligned hybrid reads mapped to the *H. erythrogramma* genome, and the remaining 42.8% mapped to the *H. tuberculata* genome (supplementary Table S3, Supplementary Material online). After quality filtering, we calculated FRIP scores as described in Methods. Of reciprocally lifted hybrid reads aligned to the *H. erythrogramma* genome, an average of 22.8% fell in peaks called on hybrids, while an average of 22.3% of hybrid reads aligned to the *H. tuberculata* genome fell in peaks called on hybrids (supplementary Table S4, Supplementary Material online).

We identified a total of 109,196 OCRs in hybrids across the 3 developmental stages examined. Of these, 102,305 OCRs (93.7%) occur within regions of the genome that are 1:1 orthologous in the 2 parental genomes. The other 6,891 OCRs (6.3%) occur within regions that are entirely absent in the genome of one or the other parental species (Davidson et al. 2022). This is consistent with previous studies comparing ATAC-seq data between species that also found a small percentage of peaks that do not lift over (Edsall et al. 2019; Davidson et al. 2022; Van Belleghem et al. 2023). Subsequent analyses involve only the orthologous set of OCRs. The overwhelming majority of orthologous OCRs in hybrids (102,305 or >99.999%) fall within OCRs called on same-species crosses, with just 4 OCRs (0.004%) present only in hybrids (Fig. 2b). The proportion of DA OCRs at each developmental stage parallels that reported in our previous study (Davidson et al. 2022) despite the sets of OCRs being independently called (i.e. they overlap but are not identical). This high degree of concordance in peak calling and parallel fractions of OCRs identified as DA indicates that the ATAC-seq data from hybrids reported here are comparable in quality to our published data from the same-species crosses (Davidson et al. 2022).

As a further check on data quality, we examined the peak height in our dataset. Reads in hybrids that map to putative promoter regions (the first peak within 500 bp upstream of the translation start site, TLS) formed more open peaks than those that map to putative distal enhancers (those between 501 and 25,000 bp from the nearest TLS; see *Methods* for further explanation) (supplementary Figs. S1 and S2, Supplementary Material online, rows 2 and 3 vs. rows 1 and 4; Welch's t-test, *P* < 2.2e-16). This result is consistent with many other studies that find core promoter regions to be generally more open than distal enhancers (e.g. Boyle et al. 2008; Klemm et al. 2019). Peaks in hybrids corresponding to putative promoter regions and enhancer regions are similar in size to those in same-species crosses (Davidson et al. 2022), suggesting that biological effects outweigh technical influences on read mapping in hybrids.

To more formally assess the primary drivers of differences in ATAC-seq reads among samples, we carried out Principal Component Analysis (PCA) of the hybrid and same-species data (Fig. 2c). Principal component (PC) 1 explained 51.85% of the variation, separating samples by species, while PC2 explained a further 10.25% of the variation, separating the hybrid reads from the same-species crosses. Along PC1, the combined hybrid reads for each sample (i.e. the sum of reads from both chromosomes) fell approximately halfway between samples from same-species crosses (Fig. 2c, solid symbols). These results indicate that both parents contribute to variation among samples. Given that chromosomes from both parents are exposed to a common molecular environment in hybrids, this result further suggests that parental genotype has a substantial influence on chromatin configuration. When reads from hybrids were separated by inferred parent-of-origin, however, each sample clustered much closer to samples from the matching same-species crosses (Fig. 2c, open symbols). This result indicates that the assignment of individual reads to parent-of-origin is generally correct. Note that a perfect overlap with the matching same-species samples is not necessarily expected even if every read is accurately assigned, as this would only occur in the absence of any *trans* genetic effects (i.e. if the molecular environment of hybrid nuclei had no influence on chromatin different from that in the same-species cross).

None of the first 4 PCs separate reads by developmental stage (Fig. 2c, supplementary Fig. S3, Supplementary Material online), suggesting that the epigenome as a whole does not change extensively across the 3 stages sampled in the *Heliocidaris* species or their hybrids. Stage-to-stage differential accessibility analysis confirmed this finding in each cross, as fewer than 0.23% of peaks were DA between stages in all crosses studied (*P*≤0.1) (supplementary Table S7, Supplementary Material online). This result is in contrast to the transcriptome, where PC1 separates samples by developmental time (Israel et al. 2016; Wang et al. 2020) (Fig. 2c of this study), as well as our previous study of chromatin accessibility (Davidson et al. 2022). While this might seem surprising, as chromatin accessibility is known to change throughout development (see *Introduction*), we note that the stages studied here represent only the latter half of the stages in our previous studies. In these studies, the PCA was only separated by stage into 2 groups: early through late cleavage (not covered here) and blastula, gastrula, and larva (the stages examined here) (Israel et al. 2016; Davidson et al. 2022). Thus, it is perhaps not surprising that we do not see variability by stage in our study. Moreover, our previous chromatin accessibility study demonstrated that there is little change in *H. erythrogramma* in the composition of open OCRs at these later 3 stages (Davidson et al 2022, Fig. 3); given that the crosses studied here involve an *H. erythrogramma* mother, we would also expect to see a similar lack of stage-to-stage differences in OCR accessibility for this hybrid due to the strong maternal influences in that species.

**Fig. 3.**
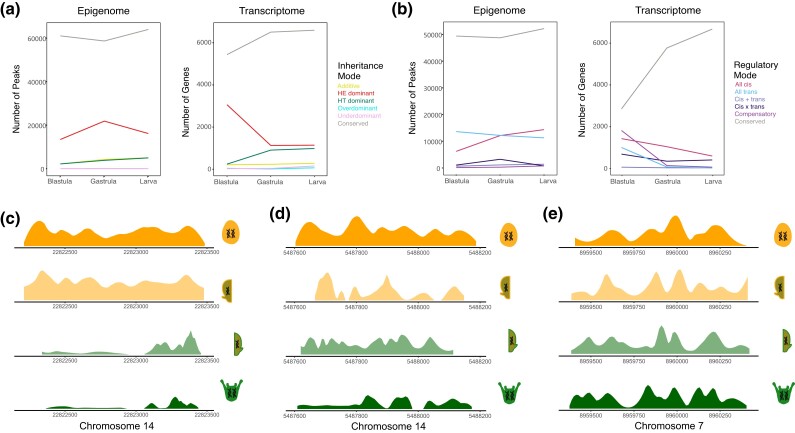
Contrasts between genetic basis for evolutionary changes in chromatin configuration and in transcript abundance, and real examples of peaks with various regulatory modes. a) Line plots of inheritance mode classification for all OCRs (left) and for all genes (right). See supplementary Fig. S6, Supplementary Material online for models of peak accessibility that exemplify these inheritance modes in the epigenome. b) Line plots of regulatory mode classification for all OCRs (left) and for all genes (right). Transcript abundance data from Wang, et al. (2020). See Fig. 2 and supplementary Fig. S4, Supplementary Material online for models of peak accessibility that exemplify these regulatory modes. c) Example from the data of browser track for a peak with a “*cis*-based” change in regulation, d) Example from the data browser track for a peak with a “*trans*-based” change in regulation. In both cases, total accessibility is shown for the same-species crosses, while for the hybrid, the browser track has been broken out into the accessibility of the 2 different alleles. e) Example from the data of browser track for a peak with “conserved” regulation, indicating no statistically significant difference in accessibility for any of the crosses and, in the case of the hybrid, no statistically significant difference in the accessibility of either allele compared to the accessibility of the respective same-species cross. Note that for the same-species crosses, total accessibility is shown, while for the hybrid, the browser track has been broken out into the accessibility of the 2 different alleles.

### Mapping Hybrid Reads to Parent-of-origin Reveals Distinct Genetic Effects

The PCA results imply that evolutionary differences in OCRs between the 2 *Heliocidaris* species have a substantial genetic component. To explore the genetic basis for divergence in chromatin configuration among species in more detail, we adapted a statistical framework originally developed for hybrid transcriptomes (Wang et al. 2020) and applied it to the set of orthologous OCRs. This approach uses a series of statistical tests to classify the genetic basis for a quantitative trait, in this case, normalized ATACseq read counts, in terms of inheritance mode (dominance effects) and regulatory mode (*cis* and *trans* effects) (supplementary Table S6, Supplementary Material online). The majority of orthologous OCRs (59.80%) are not DA between species at any of the 3 stages of development we examined (as in Davidson et al. 2022); most of these are also classified as conserved. The fraction of OCRs with conserved chromatin status is highest at blastula, the earliest stage examined (Fig. 3a and b). This is notably different from the transcriptome, which becomes progressively more similar between the 2 *Heliocidaris* species during development (Israel et al. 2016; Wang et al. 2020).

41,129 of the 102,305 orthologous OCRs (40.02%) differ in accessibility between species at 1 or more stages, and 77.1% to 88.1% (78,834, 88,664, and 90,170, respectively) of the 102,305 total OCRs could be classified according to inheritance mode depending on the developmental stage (Fig. 3a, left). At all stages, after excluding the conserved inheritance mode, the majority of these are inferred to be simple dominance effects, with just a small number of additive, underdominant, and overdominant effects. Note that the four OCRs present only in hybrids (mentioned previously) are extreme examples of overdominance. We also sought to classify the set of total OCRs by regulatory mode and found that 69.0% to 79.0% of the orthologous OCRs that differ in accessibility between species could be classified by regulatory mode (Fig. 3b, left). After excluding the conserved regulatory mode, the majority of these are inferred to be all-*cis* or all-*trans* effects (Fig. 3b) (see Fig. 1 for a visual explanation and Fig. 3c to e for examples of browser tracks for *cis, trans,* and conserved effects). A very small fraction of differential OCRs is inferred to reflect various forms of *cis*-*trans* interactions (*cis* × *trans*, *cis* + *trans*, and compensatory) (for a visual depiction of these modes, see supplementary Fig. S4, Supplementary Material online).

Most cases of differential accessibility between species are consistent with a single locus model of regulatory mode (i.e. can be explained by a single mutation). Conversely, regulatory inferences that require multiple loci (e.g. compensatory, *cis* + *trans*, and *cis*  *×*  *trans*) are relatively uncommon. These results hint at the possibility that the evolutionary divergence in chromatin states between the 2 *Heliocidaris* species often has a relatively simple genetic basis. Nonetheless, the variety of inheritance and regulatory modes suggests that a variety of molecular mechanisms can contribute to evolutionary divergence in chromatin state during early development. Moreover, a substantial number of OCRs change inheritance or regulatory mode at least once across development (supplementary Fig. S5, Supplementary Material online), suggesting that the interplay between *cis* and *trans* effects on individual OCRs can be complex. The following paragraphs examine the variety of distinct genetic effects we observed, as well as their relationship to the transcriptome.

### Maternal Dominance Effects Persist Longer in the Epigenome Than the Transcriptome

With regard to inheritance mode, many more differential OCRs were classified as maternal dominant than paternal dominant (28,263 total maternal dominant peaks vs. 7,286 total paternal dominant peaks) (for visual explanations of inheritance modes, see supplementary Fig. S6, Supplementary Material online). This ratio decreases slightly over developmental time (6.12, 5.71, 3.27 maternal:paternal at blastula, gastrula, larva). Early maternal dominance with a subsequent decrease during development is expected, as maternally provisioned regulatory molecules initially predominate in the nucleus but will be depleted over time as the zygotic genome (including paternal alleles) begins to exert an influence. Although we do observe this predicted decrease in the ratio of maternal-to-paternal dominance effects in chromatin (Fig. 3a, left), the magnitude of the drop in accessibility is much less substantial than previously reported for the transcriptome across the same developmental stages: maternal dominance of mRNA abundance is overwhelmingly more common than paternal dominance at blastula, but maternal and paternal dominance are nearly equally represented by gastrula and even closer in the larva (Fig. 3a, right).

Maternal dominance effects on chromatin outnumber all other classifications of inheritance mode put together–even in the larva, the latest developmental stage we examined. Indeed, the number of DA OCRs with maternal dominance is lowest in absolute terms at the earliest stage (blastula) and nearly doubles by the gastrula stage (13,412 peaks to 21,864 peaks). Although it may seem paradoxical for maternal effects to increase over developmental time, this is made possible by the fact that more and more OCRs become accessible during development (Davidson et al. 2022), resulting in the relative proportion of maternal dominant peaks remaining roughly stable over time despite an increase in their absolute number. These observations suggest that maternal effects on the chromatin landscape persist much longer during development than do maternal effects on transcript abundance, and are notably more extensive at later stages. This observation also indicates that dominance effects in the transcriptome do not directly parallel or reflect dominance effects in the chromatin landscape.

### Evolutionary Differences in the Epigenome are the Result of Extensive *Cis* and *Trans* Effects

Having examined the contributions of maternal and paternal genetic influences, we next considered regulatory mode. As shown in Fig. 3b, approximately equal numbers of differential OCRs are inferred to be all-*cis* and all-*trans* at the blastula stage (Fig. 3b). The number of all-*cis* OCRs rises modestly at each subsequent stage of development, slightly outnumbering all-*trans* OCRs by larva stage. Unlike dominance effects, there is no clear a priori expectation about the proportion of *cis*- and *trans*-based contributions to evolutionary divergence in chromatin status, nor how these might change during development. That said, it is not surprising to find evidence of extensive *cis*- and *trans*-based genetic influences on individual OCRs, since a variety of changes in local sequence and the *trans*-acting nuclear environment could in principle alter local chromatin configuration (Fig. 1).

The overall trends in regulatory mode during development for the epigenome are generally different from, and in some cases the opposite of, trends in the transcriptome (Wang et al. 2020; Fig. 3b). First, the number of genes with an all-*cis* change in transcription progressively decreases with developmental time, whereas it increases for chromatin. Second, the number of genes with an all-*trans* change in transcription decreases dramatically with developmental time, while in chromatin, this decrease is evident but more subtle (Fig. 3b). Third, while there are more all-*cis* than all-*trans* changes in transcription at all of the developmental changes studied, this is not the case in chromatin: instead, the number of all-*cis* changes is lower than the number of all-*trans* changes for blastula, but this ratio equals out by gastrula, and by larva there are more all-*cis* than all-*trans* changes (Fig. 3b). Finally, the 3 regulatory mode classifications that imply a genetic influence from multiple loci (*cis* + *trans*, *cis*  *×*  *trans*, and compensatory) are generally less common for chromatin than for the transcriptome (Fig. 3b). In particular, compensatory effects are moderately prevalent in the transcriptome at all 3 stages, but consistently rare for chromatin (Fig. 3b). Most dramatically, the extensive compensatory effects seen in the blastula stage in the transcriptome—which are likely due to maternal effects—are completely absent from chromatin. Alone among the multilocus inferences, *cis* + *trans* effects are similar in the transcriptome and chromatin, where they are rare at all stages examined (Fig. 3b).

### Large Differences in Chromatin Accessibility are Generally based in cis

We next considered the magnitude of genetic effects on chromatin accessibility. Specifically, we compared *cis*- and *trans*-based influences on accessibility of OCRs. We found that, at all stages, peaks with *cis*-based differences in accessibility had a greater (absolute) difference in accessibility between species than did peaks with *trans*-based differences in accessibility (supplementary Fig. S8a, Supplementary Material online, Welch's *t*-test, *P*<<0.05 at all 3 stages). Moreover, when peaks were ordered by the magnitude of these effect sizes, an average of 59% of the top 10 peaks were *cis* (supplementary Fig. S8b and c, Supplementary Material online). These results indicate that local mutations often have a greater influence on the local chromatin landscape than do *trans* effects, although there is a broad overlap.

### Genetic Basis of Differential Chromatin Predicts Differential Expression

**Fig. 4.**
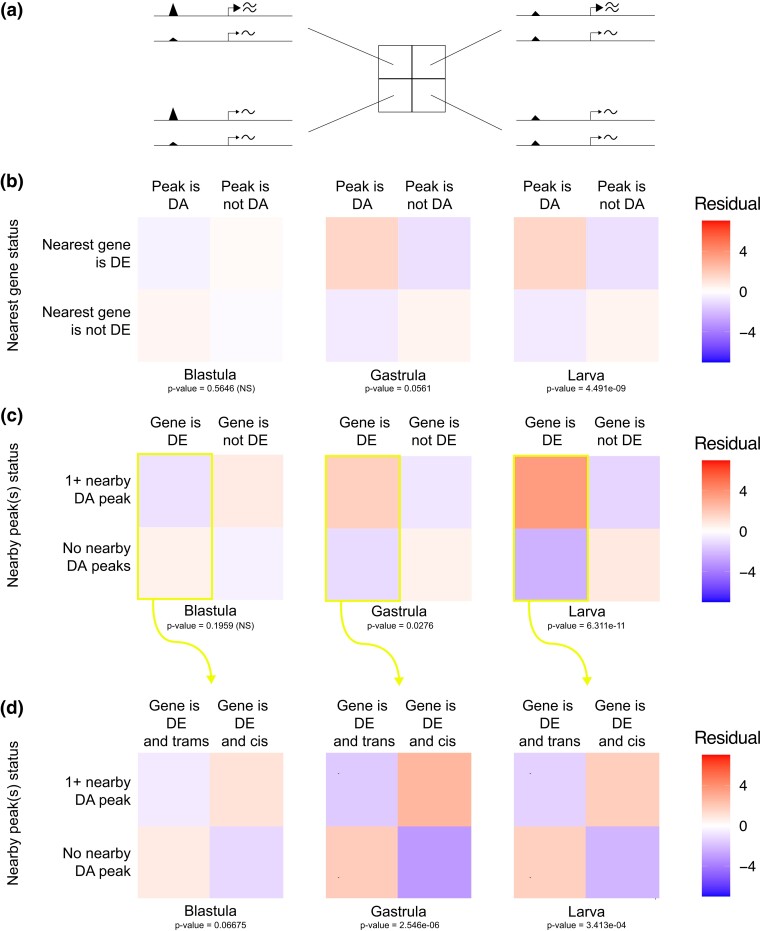
Relationship between evolutionary change in chromatin configuration and transcript abundance. Chi-squared tests for independence were used to measure the correlation between evolutionary changes in OCRs and expression of nearby genes. Heatmaps show residuals for tests carried out in 3 different contexts (see supplementary Fig. S9, Supplementary Material online for a mechanistic explanation of what each test measures). Larger residual values (darker red squares) indicate an enrichment and suggest that there are more of the given event than expected by chance. Tests were carried out separately at each of the 3 developmental stages. a) Conceptual illustration of the tests shown in b, c, and d, illustrating what each quadrant of the test represents. b) The “peaks-focused” tests ask whether the nearest gene to a DA peak is itself differential expressed more often than expected by chance. The chi-squared tests were significant (test statistic *P* < 0.1) at gastrula and larva. c) The “gene-focused” tests ask whether there is at least one DA peak within 25 kb of a DE gene more often than expected by chance. The chi-squared tests were significant (test statistic *P* < 0.05) at gastrula and larva. Note that “peaks-focused” and “gene-focused” tests are not redundant, due to the 1-to-many relationship between genes and regulatory elements. d) The “regulatory mode-focused” tests were carried out for genes that are DE between species. These tests ask whether *cis*- and/or trans-based differential expression of genes are enriched for DA peaks within 25 kb more often than expected by chance. The chi-squared tests were significant (test statistic *P* < 0.05) at gastrula and larva, with *cis*-based differential expression enriched for nearby DA peaks and *trans*-based differential expression depleted for nearby DA peaks.

Next, we turned our attention to understanding how evolutionary changes in the epigenome influence the transcription of nearby genes. Specifically, we asked whether differential chromatin accessibility is enriched near DE genes and vice-versa. (Note that these are not symmetrical tests, due to the many-to-one relationship between regulatory elements and genes.) First, we considered a “peaks-focused” perspective: if a peak is DA, is the single nearest gene more likely to be DE? Although this association was not significant at the earliest stage examined (blastula), it was significant at both gastrula and larva (Fig. 4b, supplementary Fig. S8a, Supplementary Material online, Chi-squared test for independence). Second, we considered a “gene-focused” perspective: if a gene is DE, is there any “nearby” (within 25 kb) chromatin peak that is DA? Again, for the later 2 stages, we observed that DE genes had at least one nearby DA peak more often than expected by chance (Chi-squared test for independence, Fig. 4c; supplementary Fig. S8b, Supplementary Material online). The strength of these correlations changed during development for both the “peak focused” and “gene-focused” comparisons, and was notably strongest at larva, when the zygotic genome is the most extensively transcribed and when maternal influences are the weakest among the stages examined.

We further interrogated the connection between DE genes and DA chromatin by asking whether *cis*- or *trans*-based DE genes were more associated with nearby DA chromatin. We reasoned that *cis*-based differential expression *should* be enriched near DA chromatin (since the regulation of chromatin configuration is one possible molecular mechanism contributing to evolutionary changes in transcription), while *trans*-based differential expression should not be enriched (since the inferred basis of the differential expression is located elsewhere in the genome, and thus local differences in chromatin are not the main contributors to nearby differential expression). We therefore examined OCRs near genes inferred to have *cis*-based variation in gene expression, in order to assess how much of the variation in the expression of these genes can be attributed to changes in the accessibility of nearby potential *cis*-regulatory elements. We found that genes with *cis*-based differences in expression were enriched for at least 1 DA nearby OCR at both gastrula and larva (Fig. 4d, Chi-squared test for independence, *P*≤0.1). Meanwhile, genes with *trans*-based differences in expression did not show any such enrichment (supplementary Fig. S8c, Supplementary Material online).

### Proximal and Distal Peaks Differ in Size, Regulatory Mode, and Motif Enrichment

**Fig. 5.**
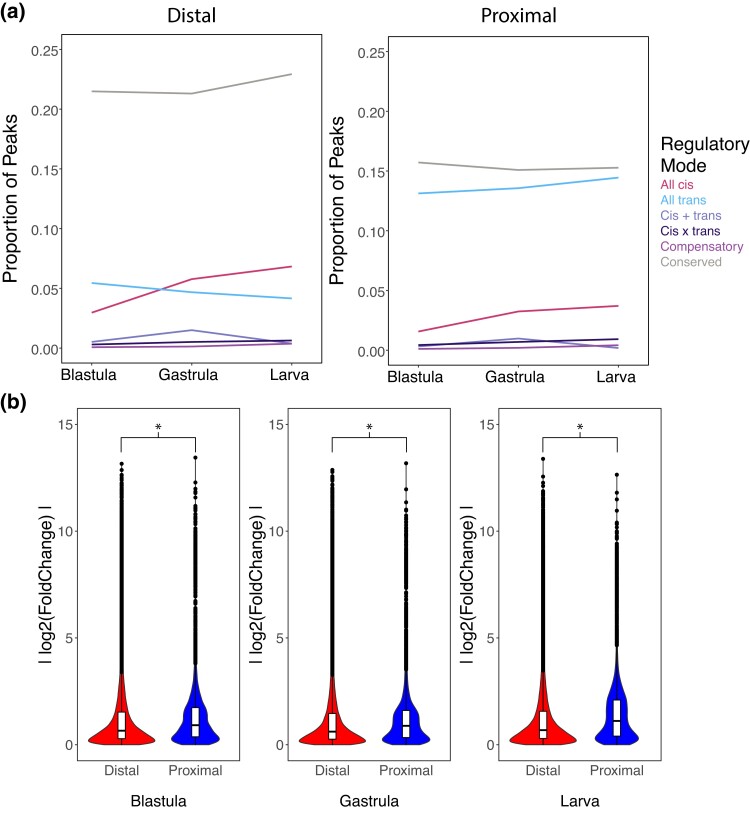
Distinct evolutionary trends in proximal and distal OCRs. a) Line plots showing the proportion of OCRs in each regulatory mode classification for proximal (<500 bp from the TLS of the nearest gene) versus distal (between 500 and 25 kb from TLS). *Trans*-based differences dominate proximal peaks, while *cis*-based differences are more common than *trans*-based differences in the (much more abundant) distal peaks. b) Violin plots contrasting the effect size for distal versus proximal peaks. At each stage, the mean effect size for proximal OCRs was significantly greater than the mean effect size for distal OCRs (Welch's *t*-test: *P* = 1.781e-12 for blastula, *P* = 8.638e-08 for gastrula, *P* < 2.23e-16 for larva). For a more detailed breakdown of the regulatory modes and effect sizes of distal peaks, see supplementary Fig. S10, Supplementary Material online.

Given that gene regulatory elements carry out diverse functions, we investigated whether OCRs show distinct evolutionary trends based on their function as core promoters and distal enhancers. We used position relative to the nearest TLS as a proxy for likely function, dividing OCRs into proximal peaks (center ≤500 bp from a TLS) and distal peaks (center >500 and <25,000 base pairs from a TLS). In total, there were 7,910 proximal peaks and 72,143 distal peaks. Proximal peaks had significantly higher rates of DA peaks than other peaks in the peak set at all 3 stages (Fisher's exact test, supplementary Table S8, Supplementary Material online), while distal peaks had significantly *lower* rates of DA peaks than other peaks in the peak set at all 3 stages (Fisher's exact test, supplementary Table S8, Supplementary Material online). When we examined the regulatory modes of these DA peaks, we found that proximal peaks are more than twice as likely to be genetically based in *trans* than distal peaks, and are also enriched for *trans* peaks relative to the proportion of *trans* peaks in the entire peak set (Chi-squared test for independence and Fisher's exact test, Fig. 5a, supplementary Table S9, Supplementary Material online). Proximal peaks also had a significantly greater effect size than distal peaks at all stages studied (Welch's *t*-test, Fig. 5b), where the effect size is defined here as the log2 of the ratio between the accessibility of the same-species peaks (as in Mattioli et al. 2020; Wang et al. 2020). These results were maintained even when the relatively large “distal” category was subdivided into more proximal and more distal regions (supplementary Fig. S10, Supplementary Material online).

To assess the molecular mechanisms controlling access to proximal and distal elements, we carried out motif enrichment using the Hypergeometric Optimization of MotifEnRichment (HOMER) motif analysis tool. Based on our previous analysis (Davidson et al. 2022), we expected to see enrichments for motifs related to pioneer factors in at least the proximal peak subset. We found that 756 motifs were enriched in proximal elements as compared to distal elements, while only 8 motifs were enriched when distal elements were in the test set (supplementary Table S10, Supplementary Material online). Among the motifs enriched in proximal elements were those for several Forkhead family transcription factors, a family containing many known pioneer factors (Zaret 2005); these included FOXK1, FOXO3, and FOXF1.

### Peaks Near Gene-regulatory Network (GRN) Genes Differ in Density, Regulatory Mode, and Motif Enrichment

**Fig. 6.**
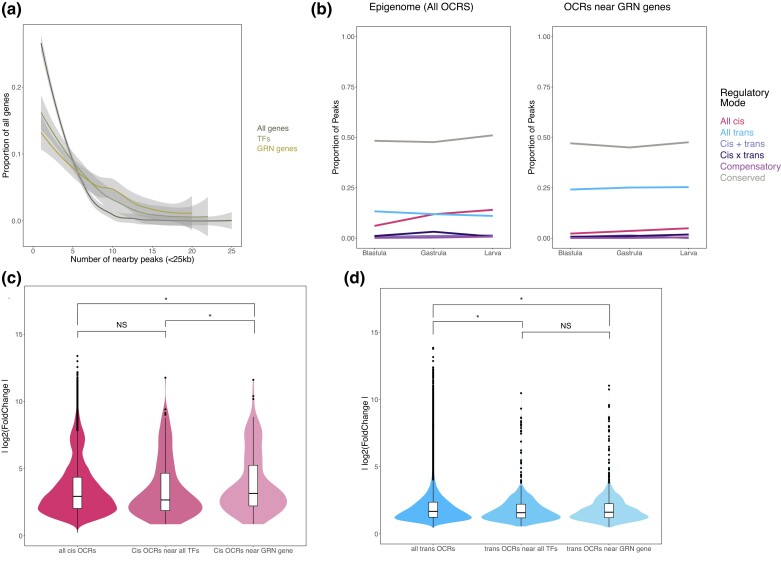
Distinct evolutionary trends in open chromatin near developmental regulatory genes. Plots present results for genes and OCRs in 3 classes of interest (“all genes”, “transcription factors” and “GRN genes”). a) Smoothed histograms of the proportion of genes with a given number of nearby peaks. The distributions for “transcription factors” and “GRN genes” were both significantly different from the distribution for “all genes” by a Kolmogorov–Smirnov test (*P*<<0.01), but not significantly different from each other. The X axis was truncated to 25 for illustration purposes (all 3 distributions are heavily right-skewed, with a tiny proportion of values >25). b) Line plots of regulatory mode classification for all peaks (left) as a proportion of the total number of peaks versus peaks within 25 kb of a GRN gene (right). c) Violin plots of effect size for *cis*-based peaks in 3 classes of interest. The mean effect size for *cis*-based peaks near GRN genes was significantly greater than the mean effect size for *cis*-based peaks near any transcription factor, and also significantly greater than the mean effect size for *cis*-based peaks in general (Welch's *t*-test and Scheffe test, both *P* < 0.05). The mean effect sizes of the latter 2 categories did not significantly differ from each other. d) Violin plots as in (c) but for *trans*-based peaks in the same 3 classes of interest. Here, the mean effect size for *trans*-based peaks near GRN genes was smaller than the mean effect size for *trans*-based peaks near any transcription factor, but the difference was not significant; however, the mean effect size for *trans*-based peaks near GRN genes *was* significantly smaller than the mean effect size for *trans*-based peaks in general (Welch's *t*-test and Scheffe test, both *P* < 0.05).

Transcriptional states in sea urchin embryos are driven by a well-defined GRN (Davidson, et al. 2002; Oliveri, et al. 2002; Saudemont, et al. 2010; Erkenbrack, et al. 2018). Previous work has established that (i) the genetic mechanisms governing the regulation of these genes differ from those controlling gene expression as a whole (Wang, et al. 2020), and (ii) putative regulatory elements near these genes are more likely to experience positive selection than the rest of the epigenomic landscape (Davidson, et al. 2022). Thus, we also examined how the open chromatin landscape near GRN genes compared to the epigenome as a whole. We found that the density distribution of peaks near (within 25 kb) of a GRN gene was significantly different from the density distribution of peaks in the entire genome (Scheffe test and Kolmogorov–Smirnov test), with GRN genes having more nearby peaks than non-GRN genes. While this difference in the epigenomic landscape may be due to a unique property of GRN genes themselves, it is also possible that it is a consequence of the fact that the vast majority of GRN genes are transcription factors. To test this possibility, we compared the density distribution of peaks near GRN genes to that of peaks near all transcription factors. We found that the GRN genes did not tend to have a greater number of nearby peaks (Fig. 6a) than the entire set of transcription factors.

To provide a richer insight into the connection between the regulation of GRN genes and the regulation of nearby regulatory elements themselves, we next queried the regulatory modes of peaks near GRN genes. These peaks were, at all 3 stages, approximately twice as likely to be *trans* compared to the epigenome as a whole (Fisher's exact test, Fig. 6b). They were also more likely to be *trans* than peaks near transcription factors as a whole (Fisher's exact test). As with the whole epigenome, *cis* peaks had a significantly greater effect size than *trans* peaks at all stages studied (Welch's *t*-test). However, *cis* peaks near GRN genes were also more open than *cis* peaks as a whole (as well as *cis* peaks near transcription factors), while *trans* peaks near GRN genes were less open than *trans* peaks as a whole (Scheffe test and Welch's *t*-test, Fig. 6c and d). Thus, peaks near GRN genes are more numerous than near the set of all genes (though not more so than near comparable genes, i.e. those encoding transcription factors not part of the GRN), and also more likely to have a *trans*-based difference in accessibility between species than the epigenome as a whole. At the same time, the magnitude of the difference in accessibility between species is markedly smaller for *trans* peaks than *cis* peaks, a contrast which is also true at the level of the entire epigenome but which is exaggerated when only peaks near GRN genes are considered.

Similarly to our analysis of proximal versus distal peaks, we compared motif enrichment in peaks that were *cis* in at least 1 stage and peaks that were *trans* in at least 1 stage to probe what molecular mechanisms might be controlling the regulation of these peaks. *Cis* peaks were enriched in 392 motifs when *trans* peaks were used as the background set. Of these motifs, 8 matched known GRN genes. When *cis* peaks were used as the background set, *trans* peaks were enriched in 43 motifs, with 15 of these motifs matching known GRN genes (supplementary Table S11, Supplementary Material online).

## Discussion

We present here the first analysis of chromatin configuration in interspecies hybrids using outbred natural populations and covering multiple stages of embryonic development. An important goal of this study was to understand the potential for evolutionary changes in DNA accessibility to influence trait evolution by modifying gene expression. While it is mechanistically plausible that changes in the accessibility of regulatory elements contribute to trait differences between species, there is little published evidence. For this to be true, 3 minimal conditions must hold: (i) chromatin status differs consistently between species, (ii) those differences influence gene expression, and (iii) they are genetically based. In prior work with the same species and developmental stages, we found that thousands of OCRs differ in accessibility between species and that these changes are concentrated in OCRs near genes encoding transcription factors and specifically on the branch leading to the derived life history (Davidson et al. 2022). These studies also found a correlation between divergence in chromatin status and divergence in the expression of nearby genes. Together, these results address the first and second conditions and further suggest that changes in chromatin accessibility contributed to the life history shift within *Heliocidaris*. In the present study, we extend evidence in support of the second condition and, for the first time, investigate the critical third condition, namely the genetic basis for evolutionary changes in chromatin accessibility and their influence on gene expression during development. Our findings can be summarized by six major themes, which we highlight below.

### Many Differences in Chromatin Accessibility are Likely to be Genetically based

Chromatin configuration can differ substantially across life history stages, cell types, and environmental conditions even within a single genotype (Thurman et al. 2012; Zhu et al. 2013; Klemm et al. 2019; Davidson et al. 2022). This raises an important question in an evolutionary context: are differences in chromatin observed between closely related species genetically based, or do they simply reflect a response to an altered nuclear environment or something in between? This question matters for understanding how natural selection operates on chromatin status, because the more direct the genetic basis is for a trait, the more readily natural selection can act on that trait. The issue is particularly acute for *Heliocidaris*, as eggs of the 2 species differ enormously in the transcripts, proteins, and metabolites that are loaded into the egg (Hoegh-Guldberg and Emlet 1997; Byrne, et al. 1999; Israel, et al. 2016; Davidson, et al. 2019). For this reason, it is plausible that differences in chromatin status between the 2 species are largely indirect effects arising from different nuclear environments, rather than arising from genetic differences. Plasticity of chromatin accessibility in response to differing nuclear environments is likely to occur via changes to the expression or localization of transcription factors that interact with chromatin—in other words, due to *trans* changes (Fig. 1). Meanwhile, the majority of differences in chromatin accessibility that are based in *cis* are likely genetic (Fig. 1). By placing chromosomes in the common nuclear environment of hybrid embryos, we were able to measure the relative contributions of *cis* versus *trans* changes to chromatin accessibility, and we found that slightly over half of the differences in open chromatin between species in *Heliocidaris* have a *cis* effect (Fig. 3b, left). Thus, a substantial number of the observed differences in chromatin accessibility between our 2 species is likely based on genetics and can be acted upon via evolutionary mechanisms. Moreover, *cis* changes by definition are local to each instance of differential chromatin—unlike *trans* changes, they cannot be explained by a change to a single upstream regulatory factor. Therefore, the widespread nature of *cis* changes in the epigenome suggests that evolutionary modifications to chromatin accessibility occurred through numerous local mutations at many different loci rather than 1 or a few modifications in upstream factors that interact with many OCRs.

### Changes in Chromatin Accessibility are Associated With Changes in Gene Expression

When considering the contribution of evolutionary changes in chromatin configuration to the evolution of gene expression, 2 important caveats should be kept in mind. First, a difference in chromatin status does not by itself indicate an influence on transcription. From a mechanistic perspective, opening chromatin is permissive rather than determinative: unless the appropriate transcription factors are present, a change in accessibility alone will not alter transcription. This is clearly illustrated by the observation that many OCRs open before the onset of transcription of any nearby gene during development, including in sea urchins specifically (Shashikant et al. 2018; Davidson et al. 2022). Second, a change in chromatin configuration is only one of several molecular mechanisms that could alter gene expression: other possibilities include a mutation in a regulatory element, a change in the presence or activity of a *trans*-acting molecule, and a variety of post-transcriptional processes.

Despite these caveats, we found a statistical association between differential chromatin status and differential gene expression at 2 of the 3 stages of development examined. The strength of this association increased over developmental time, likely reflecting the maternal-to-zygotic transition (MZT) in the mRNA pool and the progressive appearance of zygotically synthesized transcription factors during development. As expected, the statistical association is stronger for genes whose genetic basis for expression is in *cis* than for those whose genetic basis is in *trans* (Fig. 4c, supplementary Fig. S8, Supplementary Material online). Together, these results suggest that evolutionary changes in chromatin configuration contribute to some evolutionary changes in gene expression; moreover, the fact that we could detect such correlation at all, given the caveats just laid out, suggests that the role of chromatin configuration changes in changes to gene expression is not insubstantial and may, in fact, be one of the primary drivers of evolutionary differences in gene expression.

### Genetic Mechanisms Controlling Changes in Chromatin Status Show Different Patterns From Those Controlling Gene Expression

We found that the regulatory modes governing changes in chromatin status are markedly different from those controlling changes in gene expression. The number of genes with no differential expression (genes with a “conserved” regulatory mode) increased through development, indicating that the transcriptomes of the 2 species appear to converge as the embryos reach metamorphosis. However, the epigenome maintains and actually increases species-specific differences in accessibility as development progresses, as indicated by the fact that sites with a “conserved” accessibility status decrease from blastula to larva. Thus, it would appear that many differences in chromatin accessibility do not feed forward into changes in the expression of nearby genes; indeed, other studies (e.g. Connelly et al. 2014) suggest that this may be the case. However, it does appear that there is at least a loose relationship between differential accessibility and differential expression, as DA peaks are enriched near DA genes and vice versa. Moreover, genes with *cis*- but not *trans*-based differences in expression are enriched for nearby DA chromatin, suggesting that that the mechanism driving sequence-based differences in expression may be located in nearby enhancer elements. This finding also provides evidence that knowing the inferred genetic basis behind a difference in gene expression can help strengthen the ability to discover instances of differential chromatin accessibility that may be mediating the difference in gene expression.

It is also possible that these species-specific differences in accessibility do have functional relevance, but only for later stages beyond the timecourse studied here. Such a result would not be without precedent, as previous work has shown that the epigenome often becomes accessible hours before associated genes are activated (Shashikant, et al. 2018a). Overall, it appears that DA chromatin is permissive of but not always causal to changes in gene expression.

Dominance patterns also persist in the epigenome longer than in the transcriptome, as evidenced by the fact that the number of maternally and paternally dominant genes converges as development progresses, while the difference between the number of maternally and paternally dominant peaks remains fairly static over time. The MZT in sea urchins is gradual, beginning around the 16-cell stage and largely ending by the larval stage. When considering our 2 species, the MZT is somewhat delayed in *H. erythrogramma* relative to *H. tuberculata*. In contrast, we find in this study that a strong maternal influence on the epigenome lingers long after the MZT has largely eroded maternal effects on the transcriptome. Thus, this pattern also suggests that changes in chromatin status can evolve independently from changes in gene expression. The discordance between inheritance patterns in the transcriptome and the epigenome may be another example of epigenomic changes facilitating, but not necessarily directly causing, changes in gene expression, as mentioned earlier. Moreover, these persistent maternal effects at the level of the epigenome are consistent with findings in our previous work (Davidson et al. 2022) that suggest fewer active changes in chromatin status across development in *H. erythrogramma* relative to *H. tuberculata*. While more work must be done to fully understand the meaning of these results, this analysis nevertheless underscores the importance of investigating the inheritance of chromatin accessibility, as these results were not predictable based on previous work.

### *Cis* peaks have larger between-species differences in accessibility than trans peaks

When different regulatory modes were compared, we found that peaks with regulation based in *cis* had a greater average difference in accessibility (based on between-species comparisons only) than did peaks with regulation based in *trans*. This finding is similar to those seen in yeast hybrids (Ronald and Akey 2007; Connelly, et al. 2014) but is, to our knowledge, the first time this observation has been documented among species in wildtype multicellular eukaryotes. Given that *cis* changes can evolve quickly and have large influences on enhancer and promoter activity (Yona, et al. 2018; Kurafeiski, et al. 2019), we propose a model in which *cis*-based changes in general cause large increases or decreases in accessibility at a single site, while *trans* changes are more likely to be pleiotropic (Carroll 2005; Chesler, et al. 2005) and cause smaller changes in the accessibility of any individual OCR.

### *Cis* changes implicated in most striking changes to chromatin accessibility between species

We demonstrate that while both *cis* and *trans* influences have extensive effects on species-specific differences in chromatin accessibility, *cis* changes appear to exert a larger influence on the chromatin landscape overall, indicating that there is a strong genetic basis for differential chromatin accessibility. This conclusion is supported by the fact that *cis* effects occur with slightly greater frequency than *trans* effects at all 3 of the developmental stages studied, have larger average effect sizes than *trans* effects, and are overrepresented in the set of the most DA regions of the genome. Moreover, while paternally dominant changes in accessibility are rare, they are measurable and increase with developmental time, indicating that the paternal genome can and does influence chromatin accessibility during development. Combined, these results suggest that differences in chromatin accessibility between species are not due entirely, or even mostly, to differences in maternal provisioning. Nevertheless, there remains a striking role for *trans* factors in the accessibility of biologically relevant peaks, as described below.

### Chromatin Near GRN Genes Differs From the Rest of the Epigenome in Important Ways

We considered how the accessibility of peaks near GRN genes was regulated and found marked differences in regulation patterns for these peaks versus the epigenome as a whole. This was manifested in at least 3 different ways: first, GRN genes were more likely than the transcriptome overall to have nearby DA regions; second, these regions were more likely to be *trans* than the epigenome as a whole; third, despite this, the difference in accessibility across species for *cis* versus *trans* peaks near GRN genes was greatly exaggerated compared to this difference when *cis* and *trans* regions of the entire epigenome were compared. We submit that this observation is due to the level of importance of the GRN relative to the rest of the genome (Halfon 2017), leading to an exacerbated difference in accessibility between *cis* and *trans* peaks near GRN genes versus *cis* and *trans* peaks in the rest of the epigenome. Furthermore, we would expect that peaks near GRN genes would be quickly selected for or against depending on the net advantage or disadvantage they create for the organism. This would lead to the observations that *cis*-based accessibility differences near GRN genes are relatively rare, but large in magnitude where they do occur. We also noted that when motif enrichments in *cis* and *trans* peaks were compared, the set of *trans* peaks was enriched for GRN motifs relative to the set of *cis* peaks. This would suggest that peaks that are regulated in *trans* may be more likely to be influenced by changing aspects of the GRN (which are themselves often transcription factors) than are peaks regulated in *cis*.

### Concluding Thoughts

In this study, we examine how *cis* and *trans* factors contribute to patterns of chromatin accessibility in the developing embryo of two sea urchin species with markedly different life history strategies, and compare these findings to the genetic mechanisms governing gene expression during the same period of development. We find that, though differential chromatin accessibility is predictive of differential gene expression, particularly for genes with *cis*-based changes in expression, the transcriptome and epigenome are regulated very differently throughout development. *Cis* and *trans* influences both have striking effects on chromatin accessibility, with *cis*-based effects being generally larger in magnitude, enriched in distal peaks, and scattered throughout the epigenome, whereas *trans* factors are relatively smaller in effect, enriched in proximal peaks, and disproportionately influence chromatin near genes involved in the GRN. Interestingly, these regions of the genome whose accessibility is governed by *trans* factors also show evidence of enrichment for sequence-based motifs related to the GRN. Together, these findings suggest that the chromatin surrounding distinct functional classes of regulatory elements (proximal/distal peaks and peaks near GRN genes/rest of the genome) evolve in somewhat distinct ways that were previously unsuspected. Additional work will be needed to identify the underlying causes of these results. One possible influence is pleiotropy: changes in core promoter function may influence many aspects of a gene's overall expression profile, while changes in the distal enhancer function may be more often restricted to a specific aspect. Another possible factor is expression pattern complexity: developmental regulatory genes often have highly dynamic expression patterns with precise spatial boundaries and large swings in rates of transcriptional initiation over short periods of time; as a consequence, their regulatory elements may experience different functional constraints. Finally, more work is needed to measure how additional epigenetic modifications affecting accessibility (such as DNA methylation or histone modification) influence the evolution of gene expression, as we did not consider them here. Overall, our results illustrate the importance of understanding the genetic basis for evolutionary changes in the epigenome and their role in fine-tuning the regulation of gene expression.

## Methods

### Experimental Design, Animal Husbandry, and Sample Processing

We generated hybrid embryos from *H. erythrogramma* females and *H. tuberculata* males as well as both same-species crosses (Fig. 2a). (The reciprocal cross arrests as gastrulae (Raff et al. 1999), so hybrids were generated in one direction only.) We made 3 biological replicates of each cross using independent parents for each set of replicate crosses. From these crosses we collected embryos at 3 developmental stages (blastula, gastrula, larva), matching those of our previous analysis of hybrid transcriptomes (Wang et al. 2020) and a subset of stages in our previous comparative ATAC-seq study (Davidson et al. 2022). After checking the concentrations of each sample, it was determined that one hybrid library at the blastula stage was of insufficient quality to proceed to sequencing; this sample was discarded, leaving a total of 8 hybrid samples. We prepared ATAC-seq libraries from each of these hybrid samples and generated 150b p paired-end reads from these libraries. We then analyzed these along with raw reads from the same developmental stages from same-species *H. tuberculata* and same-species *H. erythrogramma* crosses, for a total of 26 samples. We obtained the same-species reads from the NCBI's Sequence Read Archive (accession number PRJNA828607) (for more on the collection and sequencing of these reads, see Davidson et al. 2022). Reads were aligned to reference genomes (Davidson et al. 2022), yielding 2,991,947 to 68,584,807 mapped reads per sample (see supplementary Tables S1 and S3, Supplementary Material online). We used macs2 (Zhang et al. 2008) to identify transposase-accessible sites with an False Discovery Rate of 5%, most of which are shared among species and present in hybrids (Fig. 2b).

Fertile *H. erythrogramma* and *H. tuberculata* adults were acquired from wild populations near Sydney, Australia, and held in ∼22 °C aquaria at the Sydney Institute of Marine Sciences. Aquaria were filled with circulating unfiltered seawater from the local bay. Cultures were produced from eggs and sperm obtained from these adults by intracoelomic injection of 0.5 KCl. A breeding design with 3 biological replicates was employed for each of the following crosse*s: H. erythrogramma* ♀*×H. erythrogramma* ♂, *H. tuberculata* ♀*×H. tuberculata* ♂, *H. erythrogramma* ♀*×H. tuberculata* ♂ (Fig. 2a). Cultures were fertilized and reared as previously described (Israel et al. 2016; Wang et al. 2020). Embryos were collected for analysis at 3 stages (blastula (12 hpf), gastrula (18 hpf), and larva (24 hpf)) and subjected to a modified version of the Omni ATAC-seq protocol (Corces et al. 2017; Davidson et al. 2022).

Hybrid sea urchins were generated as described previously (Wang et al. 2020). Briefly, *H. erythrogramma* eggs were washed in acidified sea water (pH 5) for 60 s to remove the jelly coat and then washed twice in filtered artificial seawater (FASW). Eggs were then fertilized with excess *H. tuberculata* sperm and then washed twice in FASW. Cultures were grown at 22 to 24 C with daily water changes. Because fertilization rates using this method were low (around 5%), embryos were hand-picked at the time of collection (blastula = 50, gastrula = 35, larva = 5), yielding ∼70,000 nuclei per sample.

The MinElute reaction cleanup kit (Qiagen) was used for sample purification, followed by library preparation using the Qiaquick Polymerase Chain Reaction purification kit (Qiagen) and size selection using AMPure XP beads (Beckman Coulter). Reads were sequenced on the Illumina HiSeq 4000 platform at the Duke Center for Genomic and Computational Biology. One hundred and fifty base-pair paired-end sequencing was used for the hybrid samples, while same-species samples were sequenced using a mix of 50 base-pair paired-end and single-end sequencing (see *Methods* of Davidson et al. 2022). In the interest of consistency across samples, the R2s of paired-end samples were discarded and all reads were analyzed as single-end; furthermore, all reads were trimmed to 50 bp in the trimming step. Raw reads were trimmed using TrimGalore (Krueger 2016) and the following parameter: trim_galore -q 20 –length 50 –fastqc. Next, reads were aligned to both *Heliocidaris* genomes using BBSplit, a read-binning aligner based on BBMap (sourceforge.net/projects/bbmap). Briefly, BBSplit uses BBMap to align a read to 2 genomes simultaneously, scores the alignments for mismatches, and retains the alignment with the higher score. This allows for each read in a sample to be assigned a parental genome-of-origin. As proof of principle (as well as for the sake of consistency in approach), all samples from same-species crosses were also aligned using BBSplit,with over 97% of each same-species cross's aligned reads mapping back to the “correct” parent-of-origin (supplementary Table S1, Supplementary Material online). We also created “in silico” hybrid samples as another method of testing the ability of the BBSplit tool to correctly identify the genomic origin of a read. Briefly, we aligned samples from same-species crosses to their correct genome using BBMap, subsampled a given number of aligned reads from each same-species cross, concatenated these files together, and then re-aligned the resulting in-silico hybrid sample using BBsplit. If BBsplit were perfectly able to differentiate reads from the 2 parent genomes, 50% of these in silico hybrids should have mapped to *H. erythrogramma* and 50% should have mapped to *H. tuberculata.* In actuality, an average of 50.7% mapped to *H. erythrogramma* and 49.3% mapped to *H. tuberculata* (the remaining ∼2% of reads could not be assigned confidently to 1 genome or the other). Breakdowns of parent-of-origin for the in-silico hybrids, as well as for each real hybrid sample, are available in supplementary Tables S2 and S3, Supplementary Material online.

Reads were quality-filtered using SamTools (Danecek et al. 2021) and reciprocal liftovers between the 2 reference genomes were performed using the UCSC LiftOver tool (Hinrichs et al. 2006). Briefly, reciprocal liftovers allow for all sequences to be transposed into the coordinates of just one species’ genome (in this case, *H. erythrogramma*) while minimizing the reference bias that occurs when lifting coordinates between genomes. For *H. tuberculata* reads, alignments were lifted from *H. tuberculata* coordinates to *H. erythrogramma* coordinates with a -minMatch value of 0.5; for *H. erythrogramma* reads, alignments were lifted to *H. tuberculata* coordinates and then back to *H. erythrogramma* coordinates, all with a -minMatch value of 0.5. For all samples, only reads reciprocally lifting over to the *Heliocidaris erythrogramma* genome were retained, and *Heliocidaris erythrogramma* genomic coordinates were used for all further analysis. Peaks were called for each stage across all samples from the same species (*He, Ht*, or hybrid) using macs2. Duplicate tags were also removed in the same step. The resulting narrowPeak files were combined and peaks were merged to create a master bed file, which allowed the generation of a counts table across all samples using the bedtools *multicov* function (Quinlan and Hall 2010). This counts table was the basis for further computational analyses conducted in R (version 4.0.2).

### Calculation of FRIP Scores

A standard quality control metric for ATAC-seq studies is the fraction of reads in peaks (FRIP). We calculated FRIP scores on individual samples based on established ENCODE methods (https://www.encodeproject.org/data-standards/terms/#library) using reciprocally lifted reads. However, a complication arises in dealing with reads from hybrid samples that were aligned to 2 different genomes. Since there is not (to our knowledge) a published approach for calculating FRIP scores with such reads, we opted to bin each hybrid sample into 2 “subsamples” based on the parental genome they best aligned to as described above; this approach resulted in 2 FRIP scores per hybrid sample. This method resulted in an average FRIP score of 23% for the hybrid crosses and 32% for the same-species crosses. While the FRIP scores for the hybrid crosses were lower than for the same species crosses, they were within the “acceptable” range according to ENCODE guidelines (Luo et al. 2020); moreover, the fact that these FRIP scores were obtained on essentially “half” samples suggests that the quality of reads in biologically relevant peaks was comparable for both the hybrids and the same-species crosses despite much lower read depth in the hybrid crosses. We also observed a vanishingly small number of “underdominant” peaks (see Results), which we would expect to be more common if the quality of our hybrid dataset remained poorer than that of our same-species dataset after our filtering steps (creation of a union peak set and removal of low-count reads) were performed.

### R Analysis

After using BBSplit to split the hybrid samples by the parent-of-origin of each read, there were a total of 42 samples represented in the counts table (18 parental samples + 8 hybrid samples + 16 “split” hybrid samples—see supplementary Table S5, Supplementary Material online for a breakdown of these samples and their descriptions). This counts table was imported into R (version 4.0.2) for further analysis. Low count removal was performed using edgeR's *cpm* function and omitting the split hybrid samples. Rows were required to have a minimum of 3 counts per million (CPM) in at least 1/3 of rows, leaving a total of 102,305 peaks remaining. Read counts for each peak were *vst*-transformed, and PCA was completed on these *trans*formed reads using the *prcomp* function from the “stats” R package. Inheritance and regulatory modes were defined and calculated as described in (Coolon et al. 2014; Wang et al. 2020) (see supplementary Table S6, Supplementary Material online for classification parameters).

Differential accessibility analysis was performed using the DESeq2 package (Love et al. 2014) in R. Mean expression change distributions, based on expression values from (Israel et al. 2016) were compared using 1-way analysis of variance and Scheffe's Test. These tests work for datasets that may have unequal variances, so they are appropriate for comparing distributions with large differences in the number of observations per dataset (as was the case for many of our comparisons). The GRN gene set used (supplementary Table S12, Supplementary Material online) was the same as in a previous analysis (Davidson et al. 2022), and was originally obtained from BioTapestry.org. Gene functional categories were obtained from Echinobase (www.echinobase.org) (Arshinoff et al. 2022).

When measuring the distance from an OCR to the nearest gene, the TLS was used rather than the TSS (transcription start site, as the genomes of these species lack well-annotated 5′ untranslated regions. This approach, which mirrors that used in a previous study with these genomes (Davidson et al. 2022), allows us to capture distances between OCRs and genes even when evidence about the location of the TSS is weak or absent.

### HOMER Analysis

Peaks of interest were analyzed for motif enrichments using the HOMER motif analysis tool (version 4.11) (Heinz et al. 2010), using appropriate background sets for each test as explained in the Results section.

## Supplementary Material

msad222_Supplementary_Data

## Data Availability

FASTQ Files of raw ATAC-seq reads are available on NCBI's Sequence Reads Archive (accession number PRJNA1000991). Count tables, lists of relevant gene sets for testing, and R code used to generate figures and tables are available on GitHub at https://github.com/Wray-Group-at-Duke/HybridATAC.
