# Supplementary material for: Hybrid Epigenomes Reveal Extensive Local Genetic Changes to Chromatin Accessibility Contribute to Divergence in Embryonic Gene Expression Between Species
Source: Mol Biol Evol. 2023 Oct 12;40(11):msad222. doi: 10.1093/molbev/msad222 (PMC10638671; doi:10.1093/molbev/msad222)
Supplement: msad222_Supplementary_Data [file msad222_supplementary_data.zip › SuppFigLegends_revised_clean.docx]

Supplementary Figure 1. Plots of peak size distribution for peaks <501 bp from a TLS, made using the “enrichedHeatmap” package in R.

Supplementary Figure 2. Plots of peak size distribution for peaks between 501 and 25000 bp from a TLS, made using the “enrichedHeatmap” package in R. Note that the color scale for this figure differs from that of Supplementary Figure 1.

Supplementary Figure 3. Additional principal components (PCs) do not separate ATAC-seq reads by stage. A) PCA of PC2 vs PC3. B) PCA of PC3 vs. PC4. (For PC1 vs PC2, see Figure 2).

Supplementary Figure 4. Visualizations of potential mechanisms through which different regulatory modes could occur. (Note that cis/trans mechanisms are already shown in the Main Text – see Figure 1.

Supplementary Figure 5. Alluvial plots showing inheritance and regulatory mode of peaks across developmental time. A) Regulatory mode. B) Inheritance mode. Note that, for clarity, ambiguous peaks are not displayed; therefore, peak totals from one stage to the next are not equivalent.

Supplementary Figure 6. Visualizations of potential mechanisms through which various inheritance modes could occur. (For illustrations of how different regulatory mechanisms can occur, see Figure 1 and Supplementary Figure 4). Note that, in contrast to Figure 1 and SFigure 4, here we show only the combined accessibility from both alleles rather than the individual accessibility of the two alleles present in any cross. This is a result of the way inheritance vs. regulatory mode calculations are made—while regulatory mode designations imply relative equality or inequality between alleles (both within and between crosses), inheritance mode designations rely only on comparing the relative equality/inequality of the total accessibility among crosses.

Supplementary Figure 7. Visualization of how each category contributes to the residual of the chi-square test for inequality between inheritance and regulatory modes. These tests were conducted at all three stages, and tested whether there is a statistical association between any inheritance mode classifications and any regulatory mode classifications. The test result was significant (p<0.05) at gastrula and larva, but could not be calculated for blastula. Note that this is because at blastula, the “underdominant” category does not contribute to the test statistic (because 0 peaks predicted to be classified as underdominant at this stag), and the chi-square statistic cannot make a determination of residual contribution for these squares.

Supplementary Figure 8. Effect sizes in *cis*- and *trans*-based differential open chromatin regions. (A) Violin plots of the effect size (measured as the absolute value of the log2 fold change between the same-species crosses) for *cis*- *vs* *trans*-based open chromatin regions at the three developmental stages examined. At each stage, the mean effect size for *cis*-based open chromatin regions was significantly greater than the mean effect size for *trans*-based open chromatin regions. (B) Volcano plots of open chromatin regions at each stage, stratified into the top 10% of effect sizes and the bottom 90% of effect sizes. Peaks in the top 10% for effect size are colored by regulatory classification, while peaks in the bottom 90% are light grey. (Compensatory and conserved peaks are not shown by color, as they fall within the bottom 90% of effect sizes by definition.) (C) Bar chart of the regulatory classification for peaks in the top 10% of effect size, colored as in (B).

Supplementary Figure 9. Design of chi-squared tests for correlations among evolutionary changes in chromatin configuration and transcript abundance presented in Figure 5 (main text). In the following, DA = differentially accessible and DE = differentially expressed. (A) The “peaks-focused” test (Figure 5A). We considered all DA peaks, and asked if, for each peak, the nearest gene was DE. Under the null hypothesis H_0_ (no connection between chromatin and transcription), we expect to see no enrichment for the nearest gene being DE. Under the alternative hypothesis H_1_ (a connection between chromatin and transcription), we expect to see an enrichment for the nearest gene being DE. (B) The “genes-focused” test (Figure 5B). We considered all DE genes, and asked if at least 1 peak within 25 kb of that gene’s translation start side was DA. Under the null hypothesis H_0_ (no connection between chromatin and transcription), we expect to see no enrichment for DA peaks near that gene. Under the alternative hypothesis H_1_ (a connection between chromatin and transcription), we expect to see an enrichment for DA peaks near that gene. (C) The “regulatory mode-focused” test (Figure 5C). For these tests, we began with the set of DE genes from Figure 5B) and subset them into those with expression differences based in *cis* or *trans*; we then asked whether *cis*- and/or *trans*-based DE genes had nearby DA peaks more often than expected by chance. The rationale is that *cis*-based DE is due to a local mutation that might act by altering chromatin configuration (although it could also act by altering transcription factor binding), whereas *trans*-based DE is not due to a local mutation and should not show any correlation with local chromatin. Under the null hypothesis H_0_ (no connection between chromatin and transcription), we expect to see no enrichment for nearby DA peaks for either *cis*- or *trans*-based DE genes. Under the alternative hypothesis H_1_ (a connection between chromatin and transcription), we expect to see an enrichment for nearby DA peaks for *cis*-based DE genes and possibly a depletion for nearby DA peaks for *trans*-based DE genes.

Supplementary Figure 10. “Distal” and “proximal” peak frequency and effect sizes when definition of “distal” is broken down into finer distance scales. A) Line plots illustrating that the proportion of *trans* peaks in distal regions is greatest for the more “proximal” of the distal peaks (upper panel) and lowest for the most “distal” of the distal peaks (bottom panel). The proportion of peaks regulated in *trans* for the second-most distal (middle panel) and most distal (bottom panel) of the distal peaks was significantly lower than the proportion of peaks regulated in *trans* for the original set of proximal peaks (data not shown, but compare blue lines in middle and bottom panels to blue line in Figure 7B of the main text—note difference in scale on y-axis). B) Violin plots contrasting the effect size for distal *vs* proximal peaks for each of these finer distance scales. For the second-most (middle panels) and most (bottom panels) “distal” of the distal peaks, the mean effect size for proximal open chromatin regions was significantly greater than the mean effect size for distal open chromatin regions, as in the original test (Welch’s t-test, p<<0.05 for all tests). However, for the most “proximal” of the distal peaks (upper panel), the difference in effect size was only significant for the larval stage (Welch’s t-test, p=4.39e-11).
