# Supplementary material for: Hybrid Epigenomes Reveal Extensive Local Genetic Changes to Chromatin Accessibility Contribute to Divergence in Embryonic Gene Expression Between Species
Source: Mol Biol Evol. 2023 Oct 12;40(11):msad222. doi: 10.1093/molbev/msad222 (PMC10638671; doi:10.1093/molbev/msad222)
Supplement: msad222_Supplementary_Data [file msad222_supplementary_data.zip › Table legends.docx]

SUPPLEMENTARY TABLES

Table S1. Read alignment statistics for same-species crosses using BBsplit to align to both parental genomes.

Table S2. Read alignment statistics for simulated “hybrid” reads generated by combining reads from both same-species crosses and then aligning these to both parental genomes using BBsplit.

Table S3. Read alignment statistics for real hybrid crosses using BBsplit to align to both parental genomes.

Table S4/ Hybrid FRIP scores generated using the methodology recommended by ENCODE (calling peaks individually on each sample). For each hybrid sample, peaks were called separately on the fraction aligning to each parental genome, yielding two separate FRIP scores.

Table S5. Sample descriptions, including sample name and stage, for samples used in R analysis. These do not include samples used to build the set of peaks that were later removed because they were PCA outliers or to balance statistical power.

Table S6. Inheritance and regulatory mode classifications.

Table S7. Number of peaks that change accessibility between stages for each cross in the experimental design. These were calculated after removing low counts from the analysis.

Table S8. Number of OCRs by regulatory mode, broken down by proximity to gene (distal vs. proximal; see *Methods* for details). Regulatory modes highlighted in yellow are those for which the peaks were differentially expressed between same-species crosses; these are summed together on the right for the purposes of the chi-square tests.

Table S9. Number of proximal and distal peaks called as *trans* at each stage of the experimental design, and results of chi-square tests on these statistics.

Table S10A. Top motifs (generated by a HOMER analysis) enriched in proximal peaks when distal peaks were used as the background set. Cells in yellow are motifs associated with the GRN.

Table S10B. Top motifs (generated by a HOMER analysis) enriched in distal peaks when proximal peaks were used as the background set. Cells in yellow are motifs associated with the GRN.

Table S11A. Top motifs (generated by a HOMER analysis) enriched in *cis* peaks near GRN genes, when *trans* peaks near GRN genes were used as the background set. Cells in yellow are motifs that are themselves associated with the GRN.

Table S11B. Top motifs (generated by a HOMER analysis) enriched in *trans* peaks near GRN genes, when *cis* peaks near GRN genes were used as the background set. Cells in yellow are motifs that are themselves associated with the GRN.
