# Supplementary material for: Hybrid Epigenomes Reveal Extensive Local Genetic Changes to Chromatin Accessibility Contribute to Divergence in Embryonic Gene Expression Between Species
Source: Mol Biol Evol. 2023 Oct 12;40(11):msad222. doi: 10.1093/molbev/msad222 (PMC10638671; doi:10.1093/molbev/msad222)
Supplement: msad222_Supplementary_Data [file msad222_supplementary_data.zip › Fig S4_addtlregmechansims.pdf]

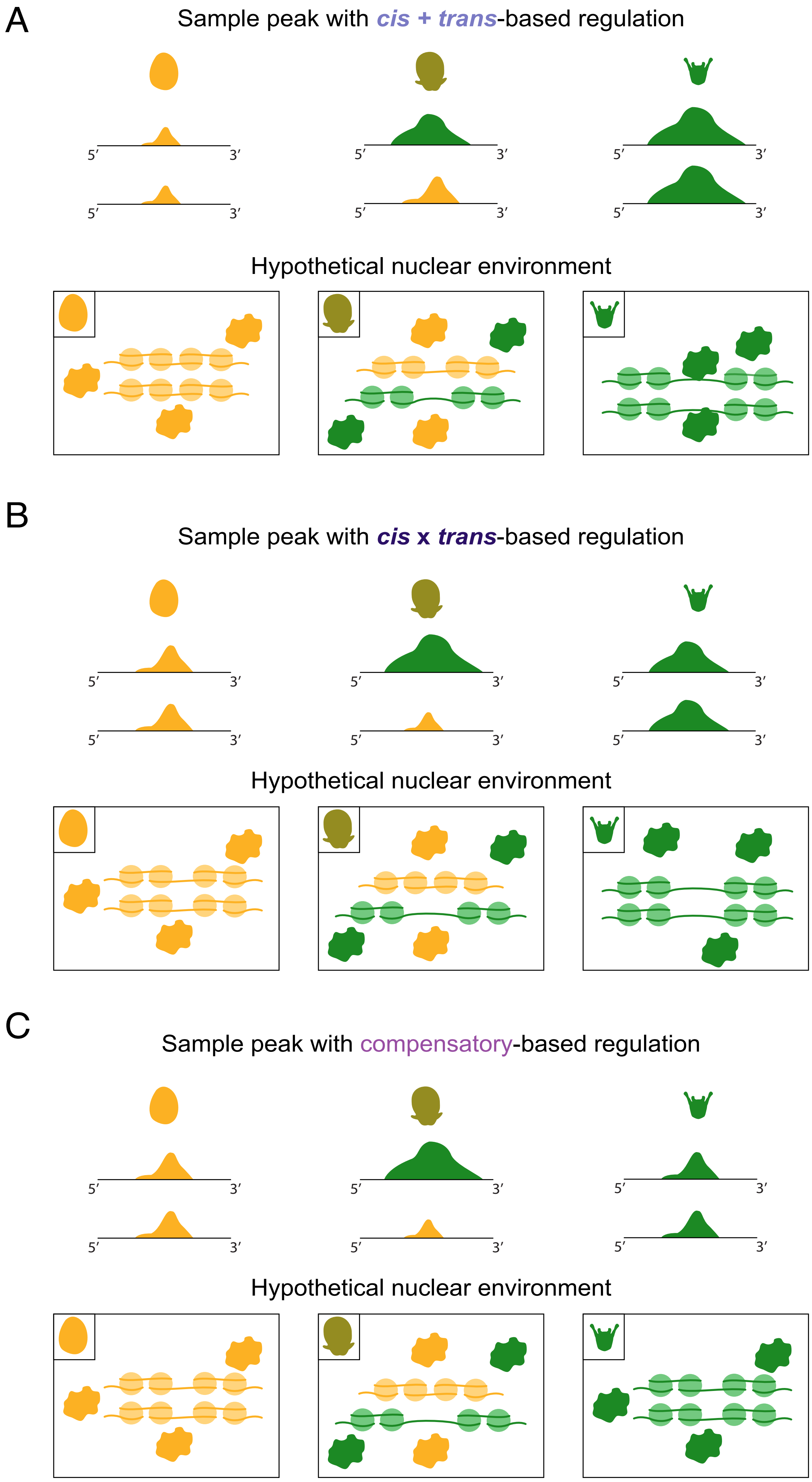
A
Sample peak with cis + trans-based regulation
5′
3′
Hypothetical nuclear environment
B
Sample peak with cis x trans-based regulation
5′
3′
Hypothetical nuclear environment
C
Sample peak with compensatory-based regulation
5′
3′
Hypothetical nuclear environment
