# Supplementary material for: Hybrid Epigenomes Reveal Extensive Local Genetic Changes to Chromatin Accessibility Contribute to Divergence in Embryonic Gene Expression Between Species
Source: Mol Biol Evol. 2023 Oct 12;40(11):msad222. doi: 10.1093/molbev/msad222 (PMC10638671; doi:10.1093/molbev/msad222)
Supplement: msad222_Supplementary_Data [file msad222_supplementary_data.zip › Fig S6_inheritancemodes.pdf]

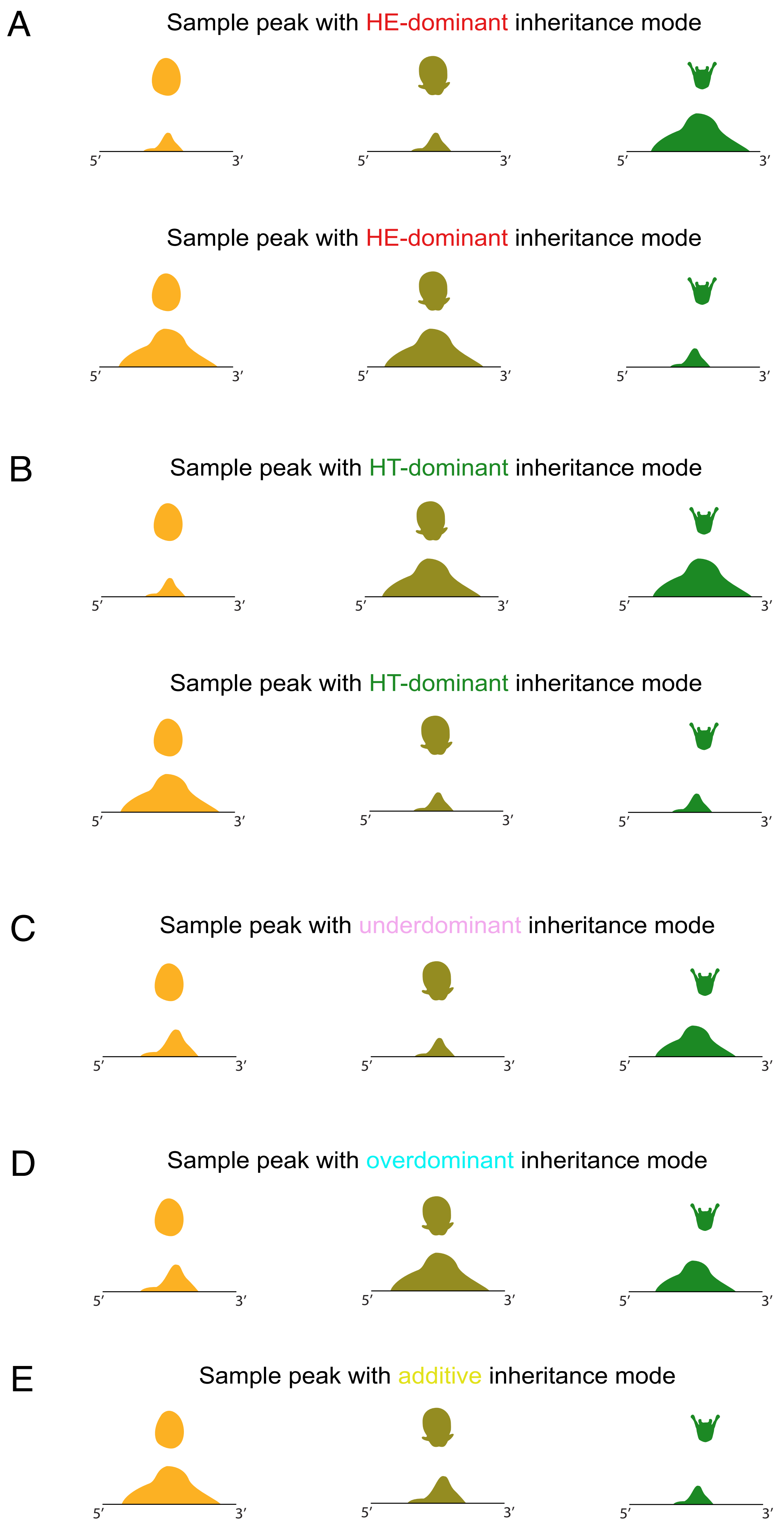
A
Sample peak with HE-dominant inheritance mode
5′
3′
Sample peak with HE-dominant inheritance mode
5′
3′
B
Sample peak with HT-dominant inheritance mode
5′
3′
Sample peak with HT-dominant inheritance mode
5′
3′
C
Sample peak with underdominant inheritance mode
5′
3′
D
Sample peak with overdominant inheritance mode
5′
3′
E
Sample peak with additive inheritance mode
5′
3′
