# Supplementary material for: Hybrid Epigenomes Reveal Extensive Local Genetic Changes to Chromatin Accessibility Contribute to Divergence in Embryonic Gene Expression Between Species
Source: Mol Biol Evol. 2023 Oct 12;40(11):msad222. doi: 10.1093/molbev/msad222 (PMC10638671; doi:10.1093/molbev/msad222)
Supplement: msad222_Supplementary_Data [file msad222_supplementary_data.zip › Fig S9 - schematic of peaks based vs gene based gene-peak connection.pdf]

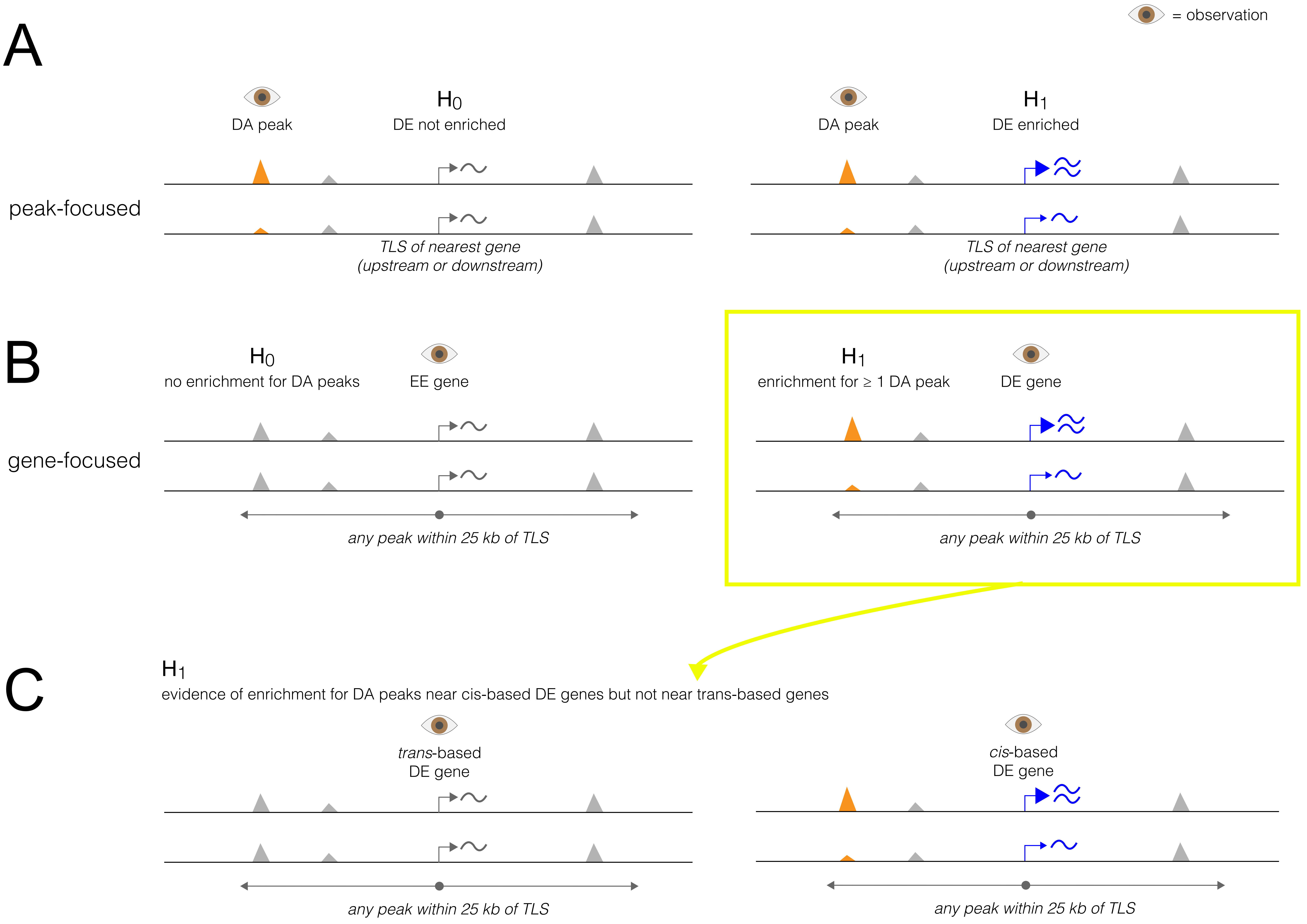
= observation
A
peak-focused
DA peak
H0
DE not enriched
TLS of nearest gene
(upstream or downstream)
DA peak
H1
DE enriched
TLS of nearest gene
(upstream or downstream)
B
gene-focused
H0
no enrichment for DA peaks
EE gene
any peak within 25 kb of TLS
H1
enrichment for ≥ 1 DA peak
DE gene
any peak within 25 kb of TLS
C
H1
evidence of enrichment for DA peaks near cis-based DE genes but not near trans-based genes
trans-based
DE gene
any peak within 25 kb of TLS
cis-based
DE gene
any peak within 25 kb of TLS
