# Supplementary figures and images for: Hybrid Epigenomes Reveal Extensive Local Genetic Changes to Chromatin Accessibility Contribute to Divergence in Embryonic Gene Expression Between Species
Source: Mol Biol Evol. 2023 Oct 12;40(11):msad222. doi: 10.1093/molbev/msad222 (PMC10638671; doi:10.1093/molbev/msad222)

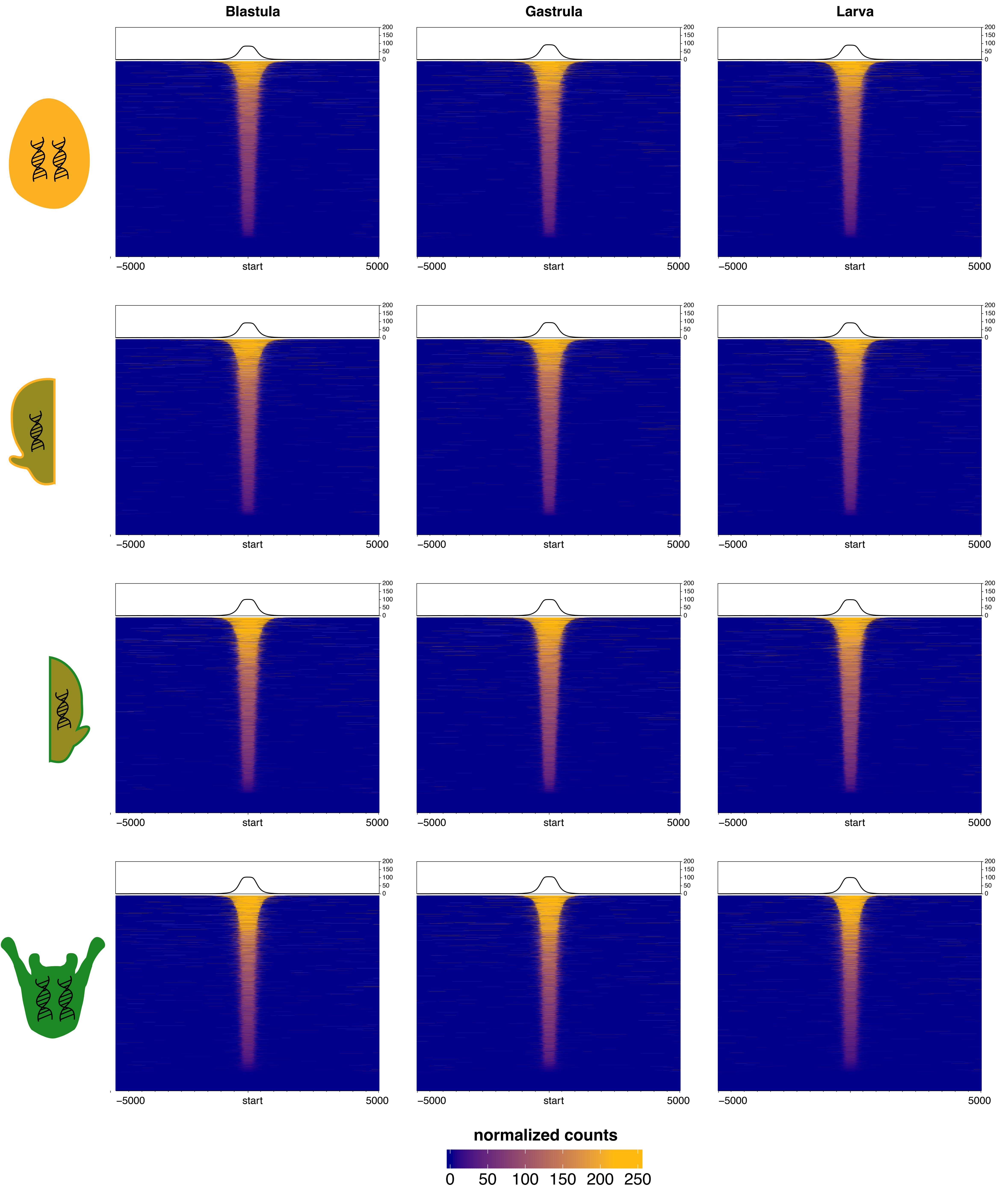

Supplement: msad222_Supplementary_Data [file msad222_supplementary_data.zip › Fig S1 edited - enrichedheatmaps_proximal.pdf]

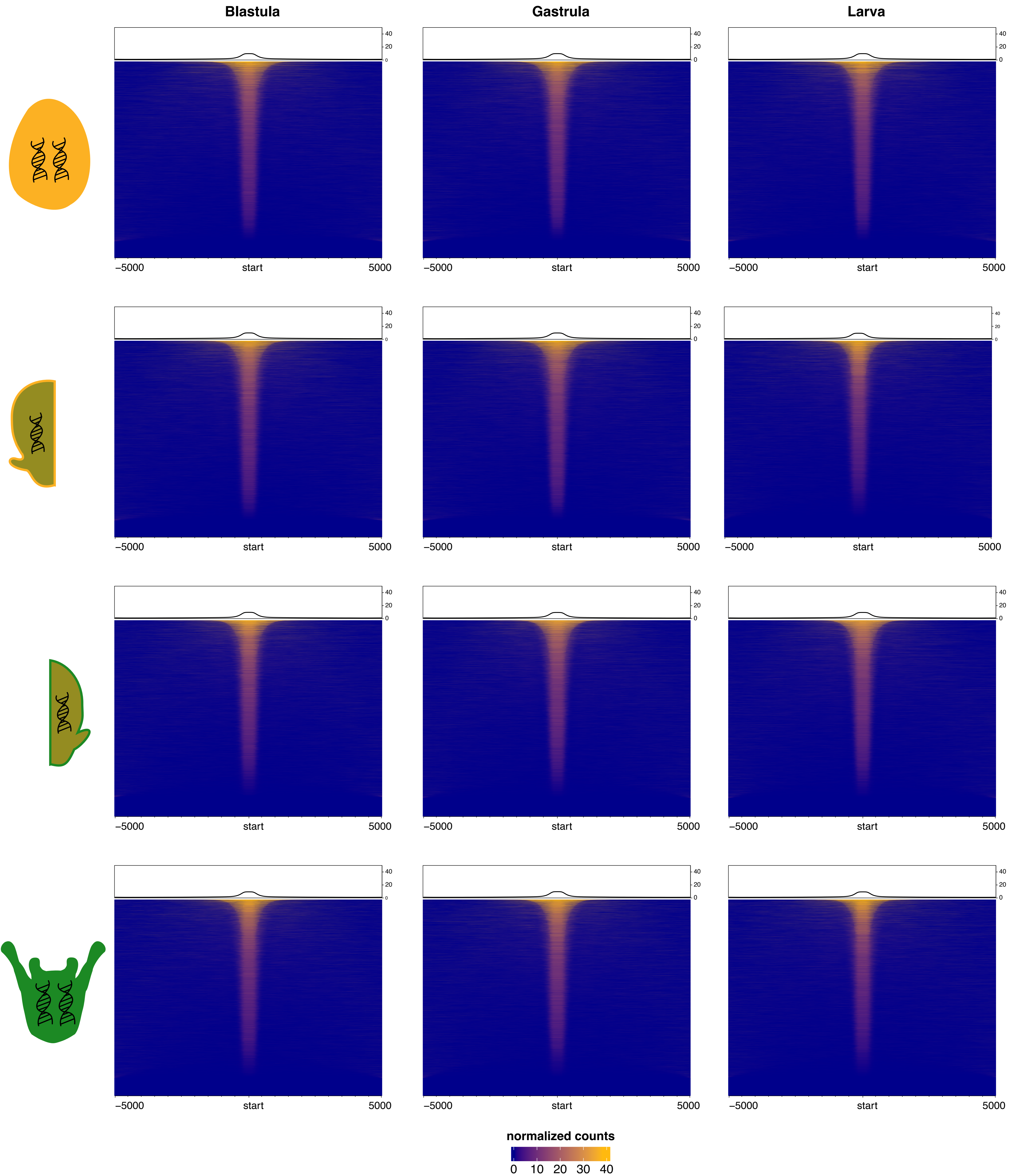

Supplement: msad222_Supplementary_Data [file msad222_supplementary_data.zip › Fig S2 edited - enrichedheatmaps_distal.pdf]

# A

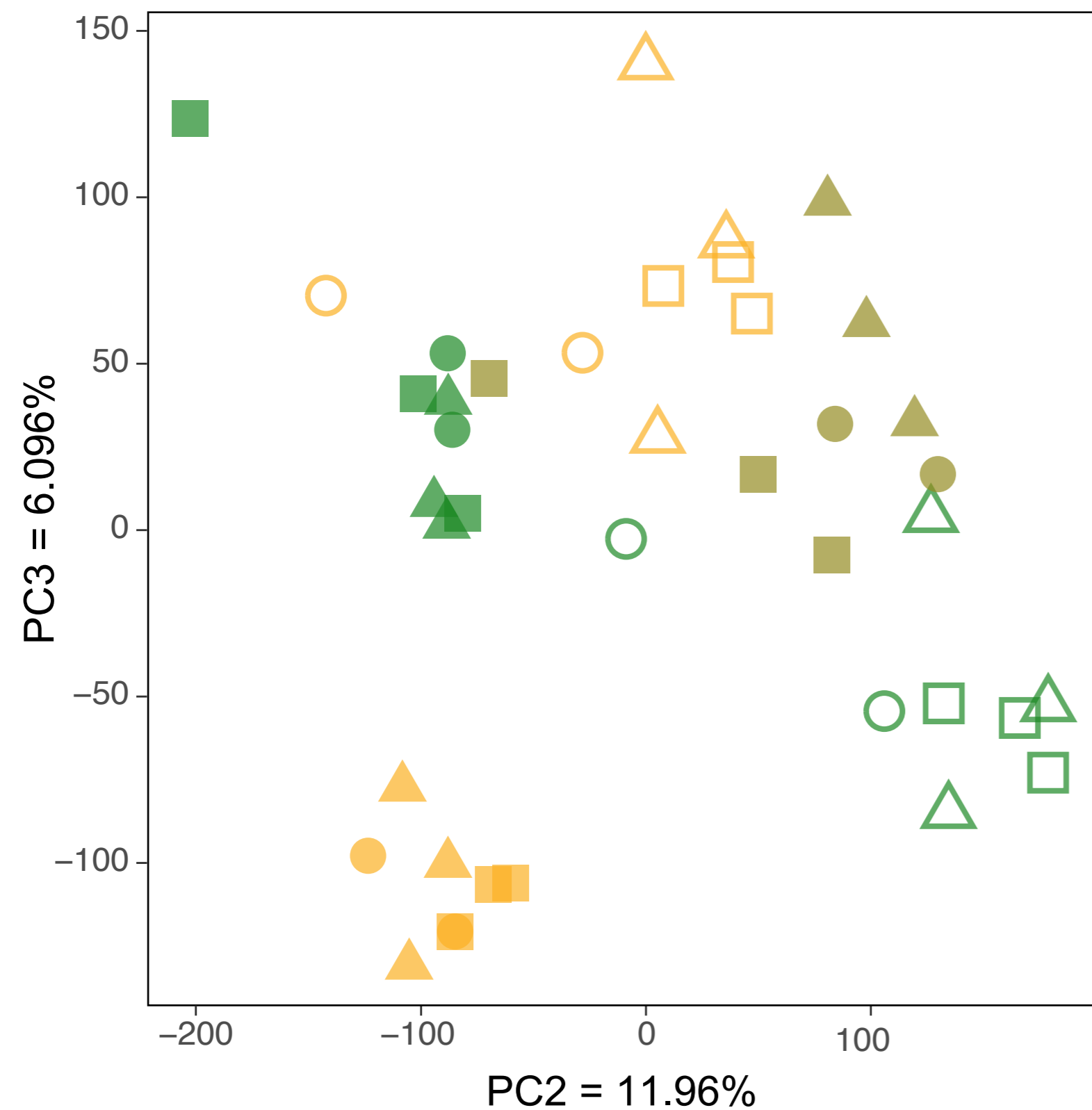

# B

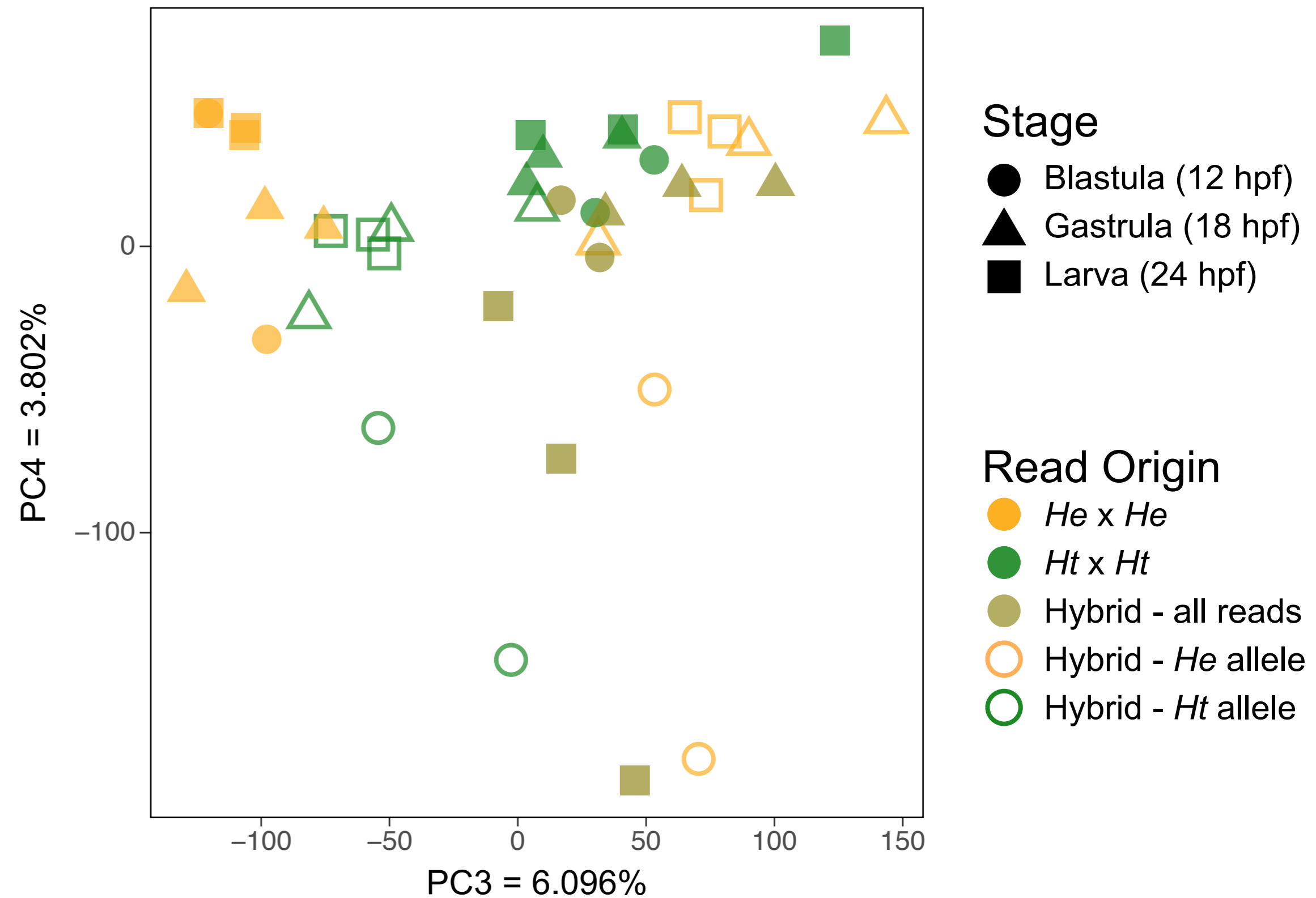

Supplement: msad222_Supplementary_Data [file msad222_supplementary_data.zip › Fig S3 - additionalPCAs.pdf]

A

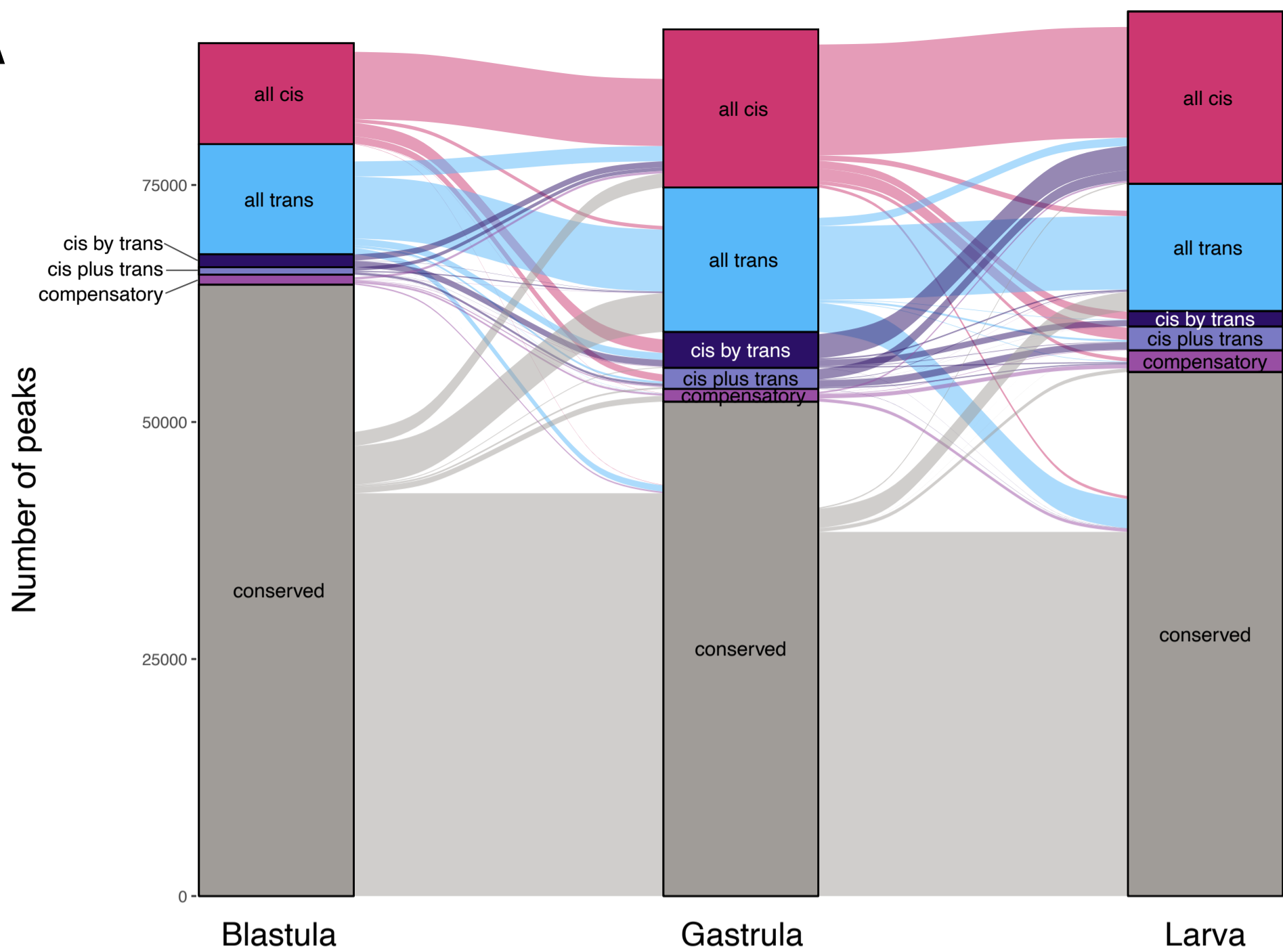

B

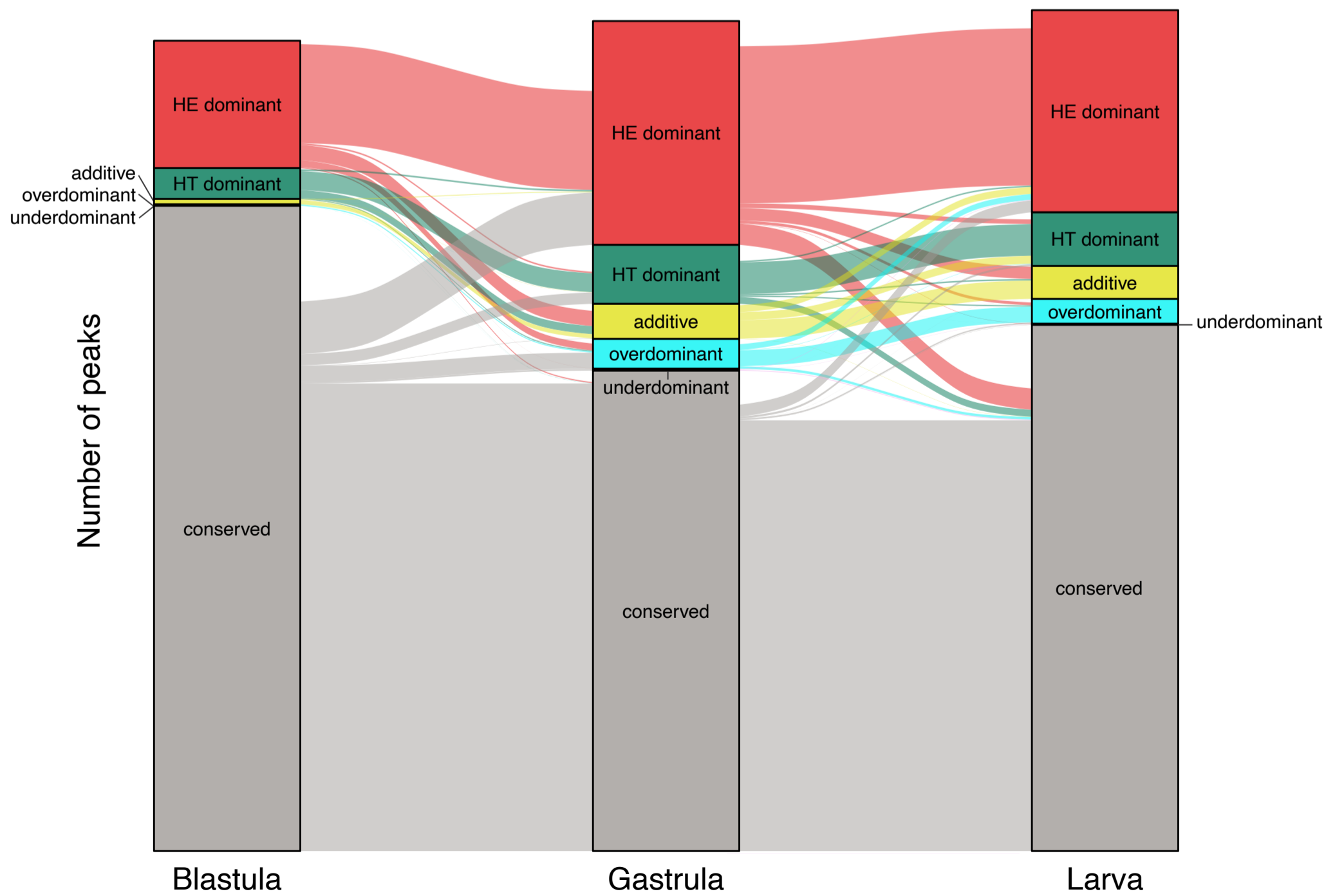

Supplement: msad222_Supplementary_Data [file msad222_supplementary_data.zip › Fig S5 - Alluvial plots.pdf]

# Blastula

p-value = NA

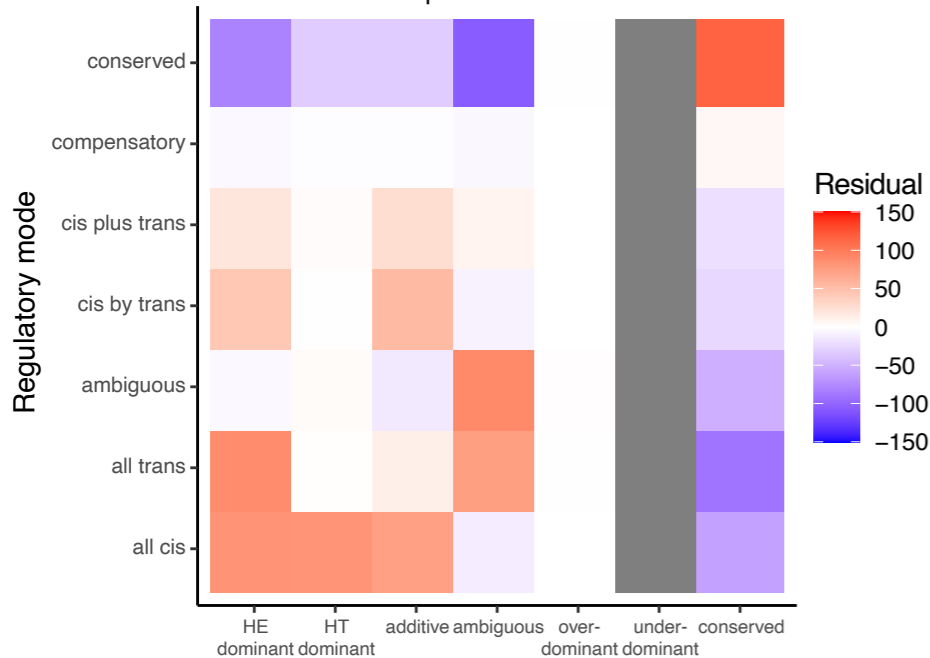

# Gastrula

p-value < 2.2e10^-16

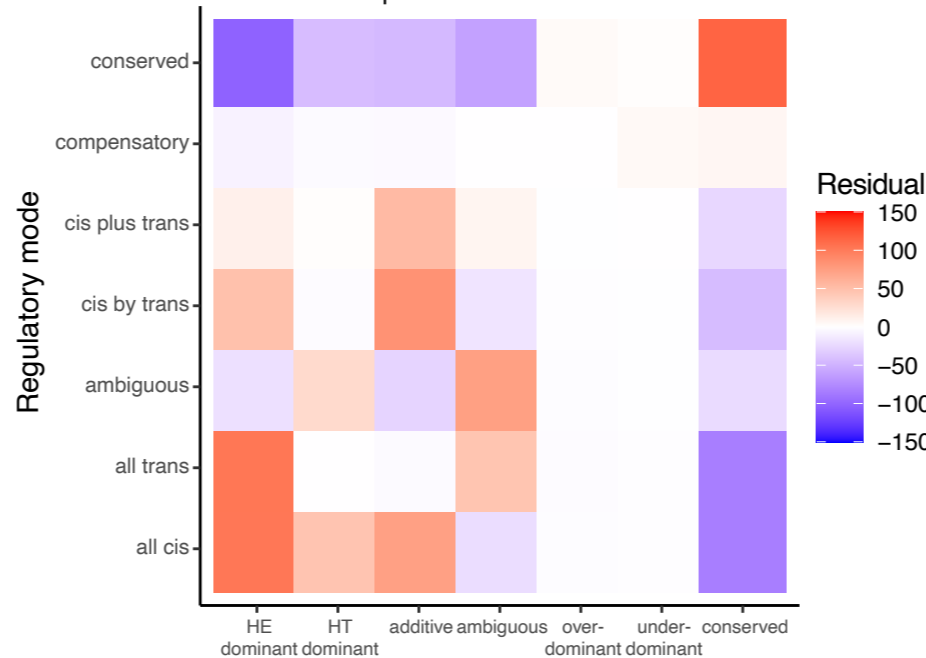

# Larva

p-value < 2.2e10^-16

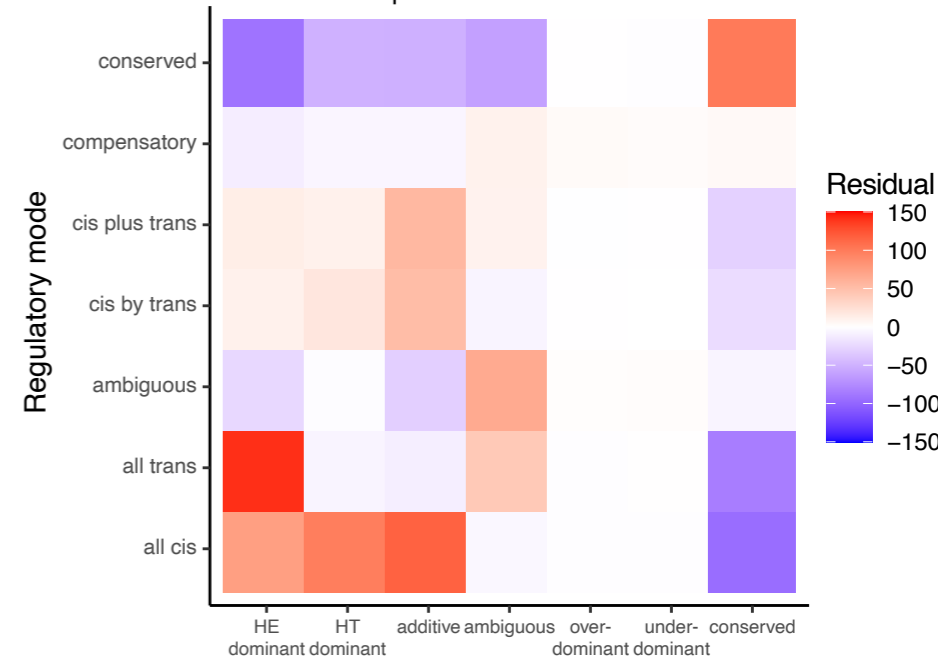

Supplement: msad222_Supplementary_Data [file msad222_supplementary_data.zip › Fig S7- Inh by CT chi square.pdf]

**A**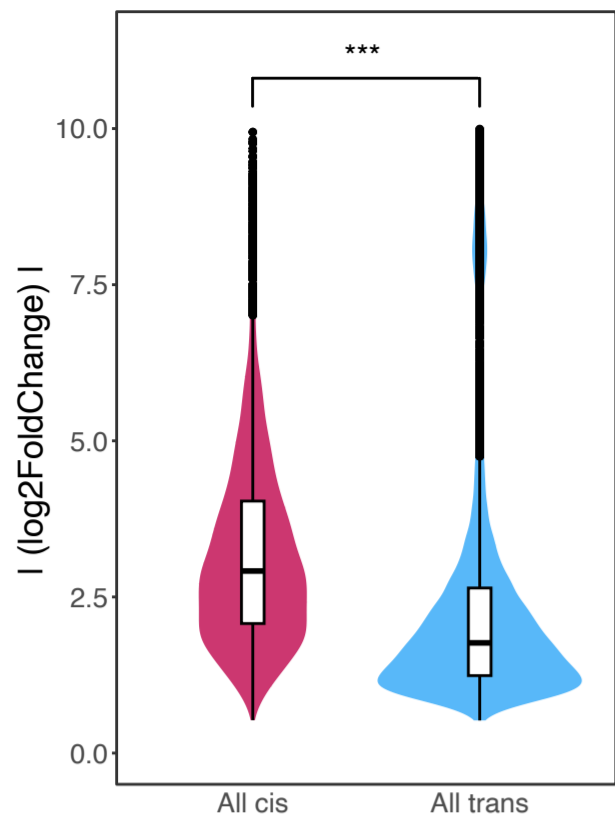**B**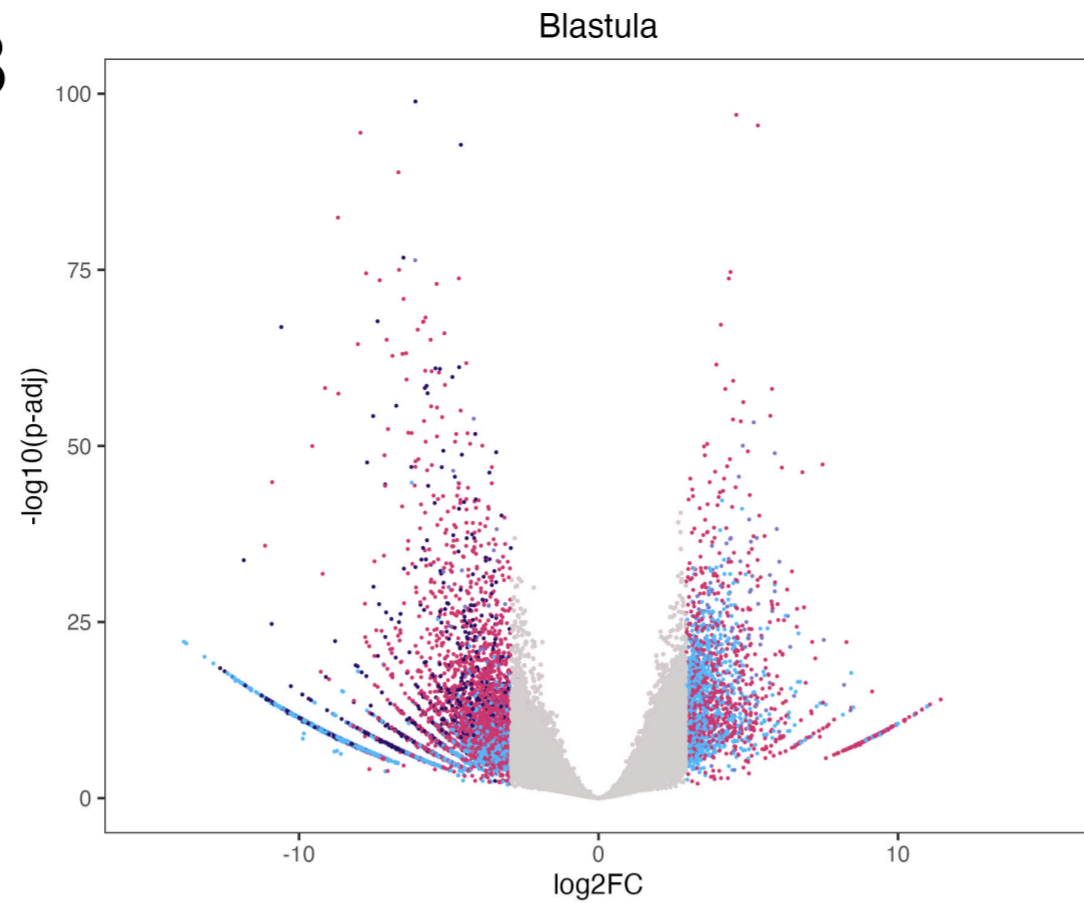**C**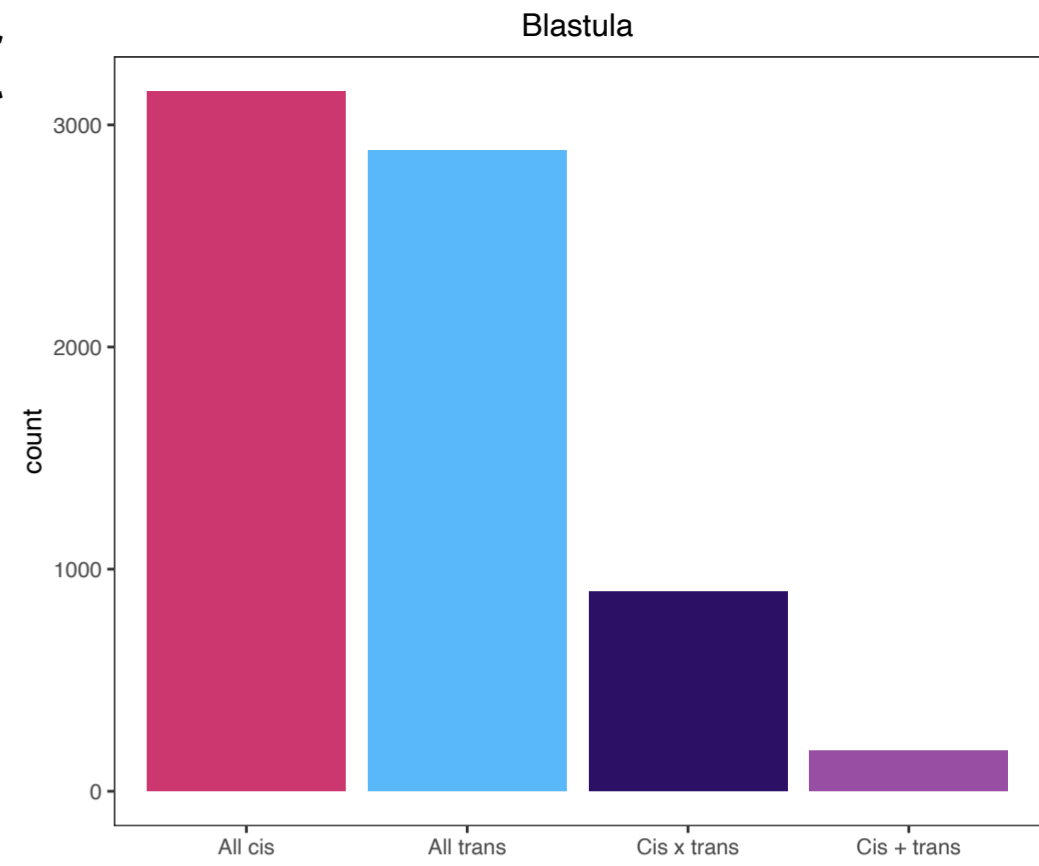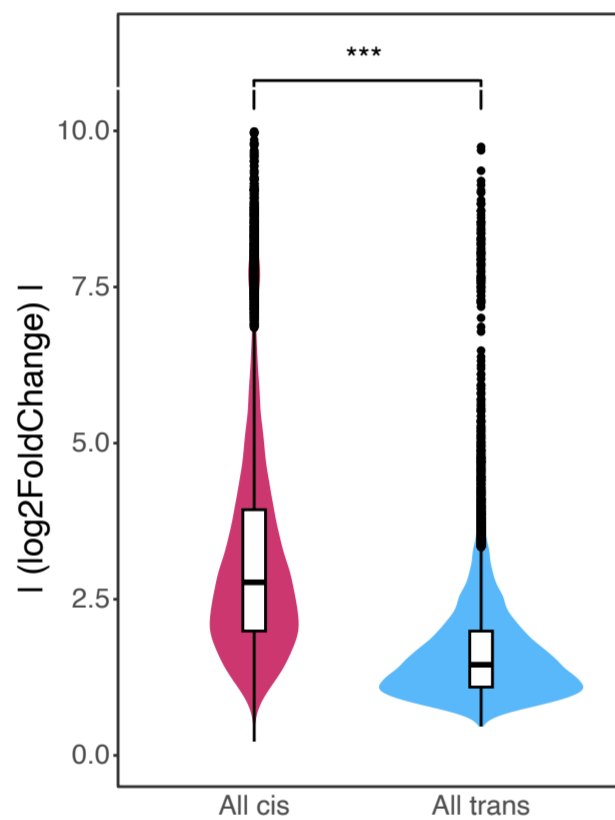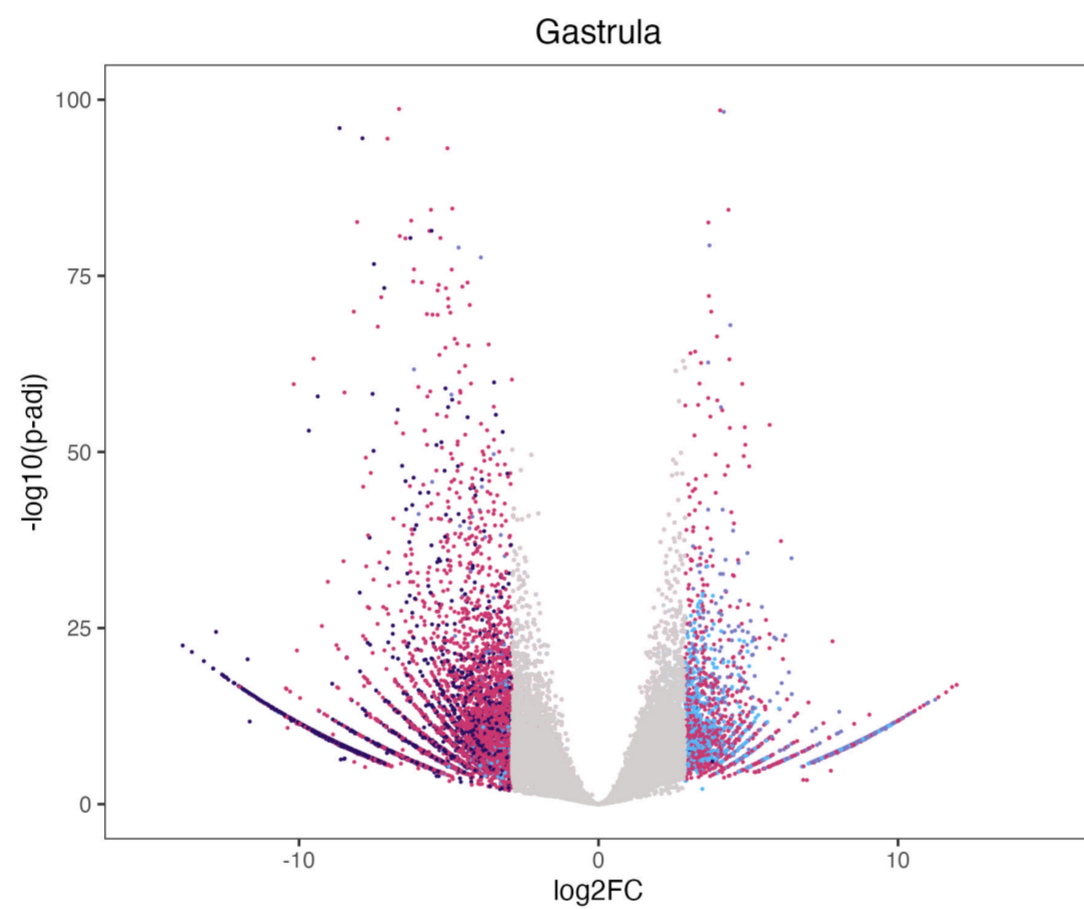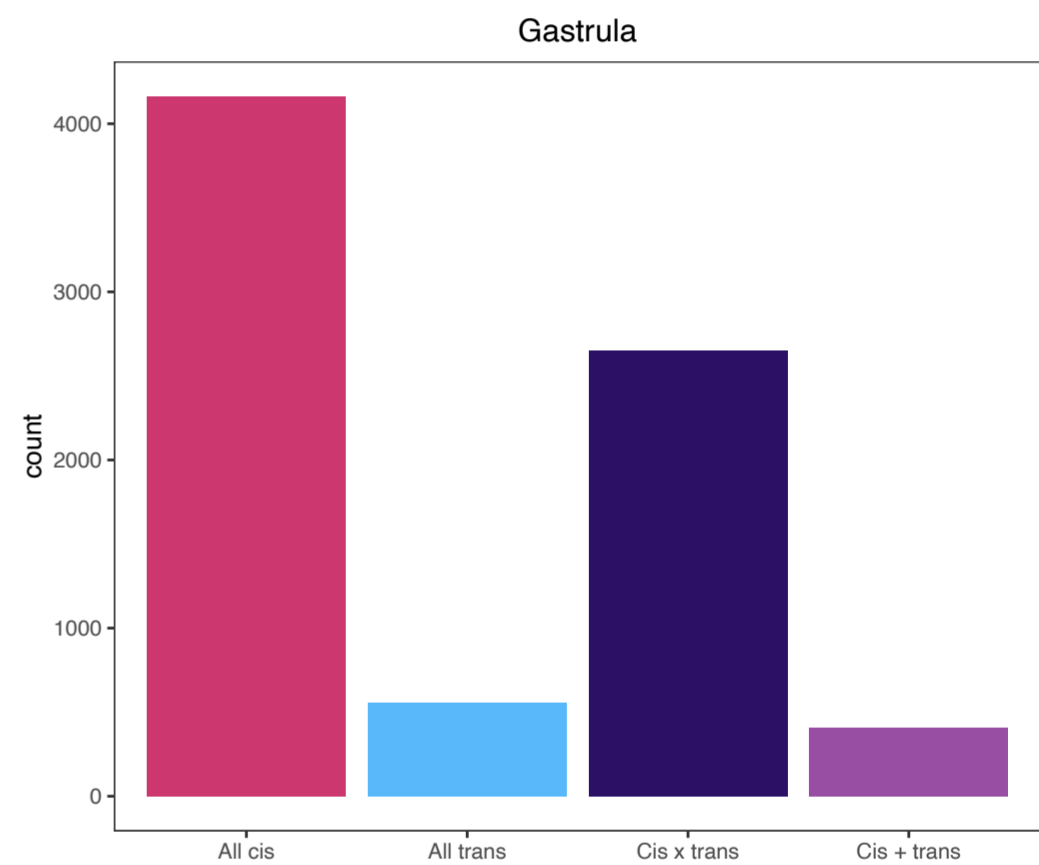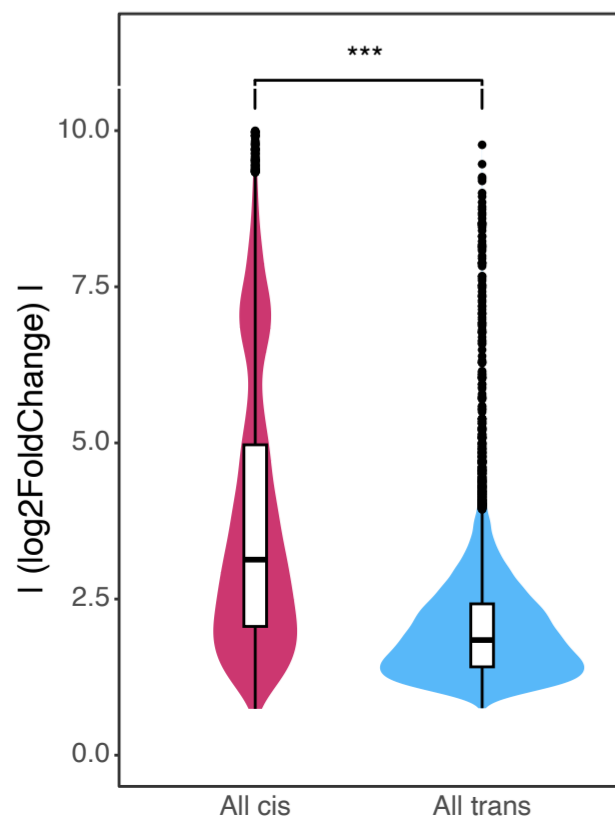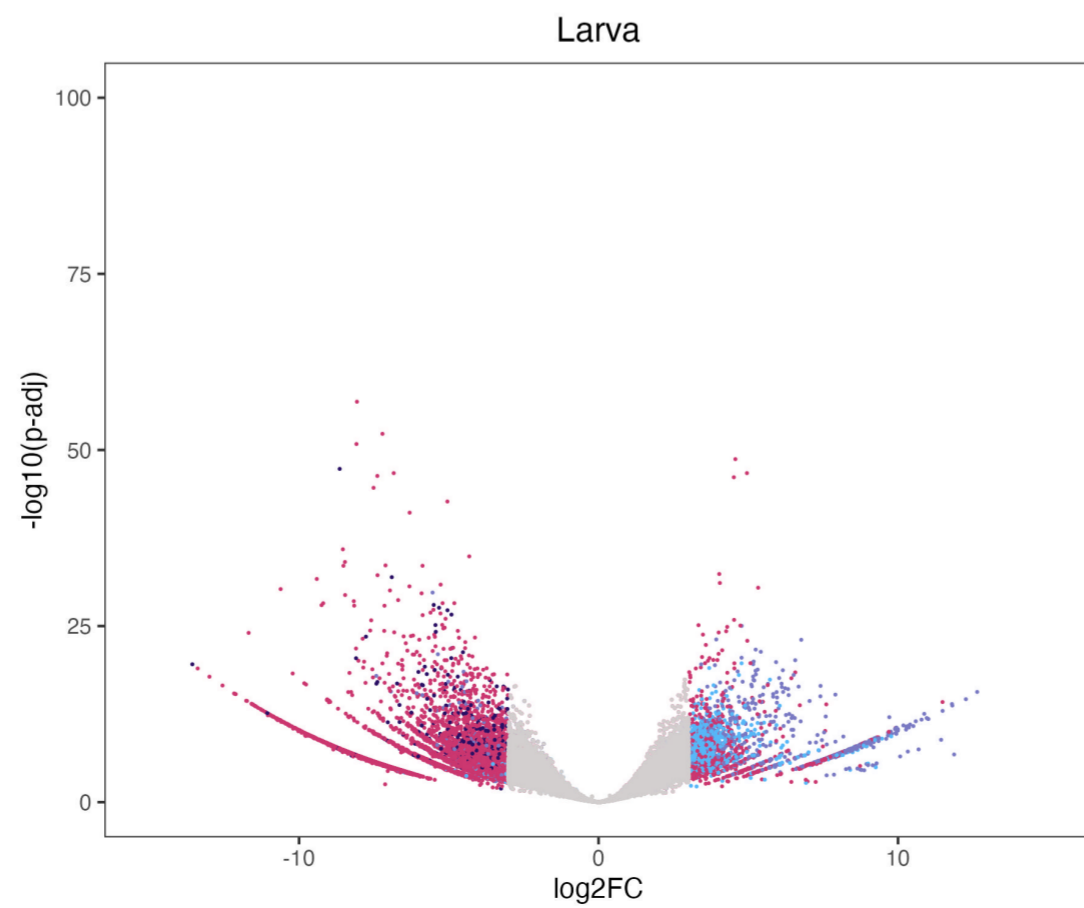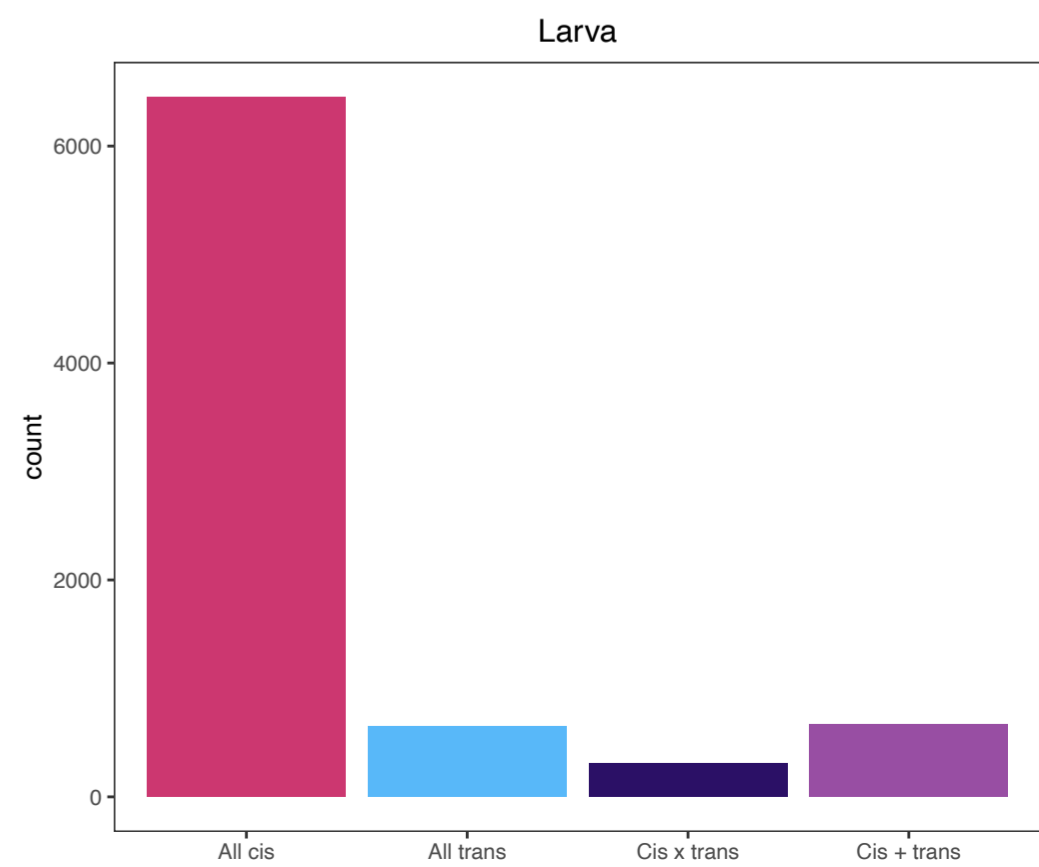

Supplement: msad222_Supplementary_Data [file msad222_supplementary_data.zip › Fig S8 - top 10 pct are mostly cis.pdf]

**A**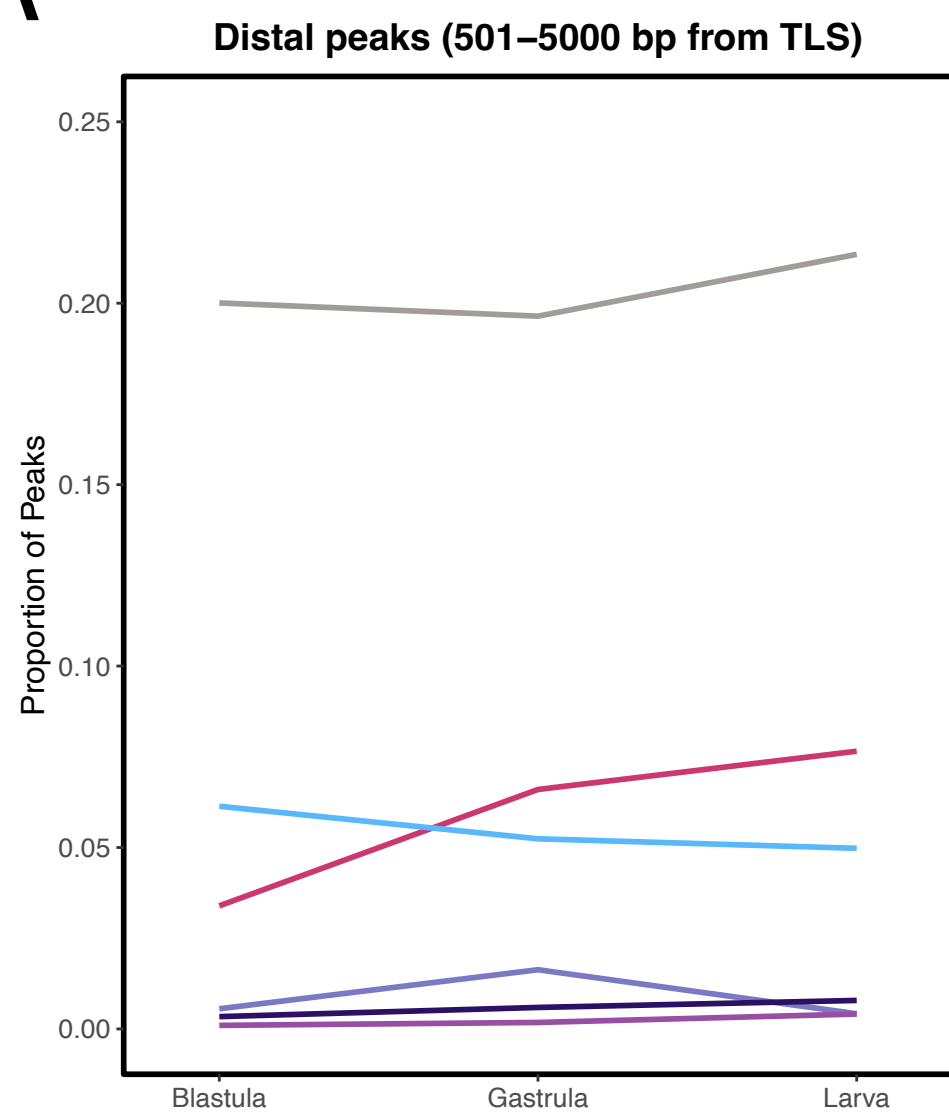**B****Distal peaks (501–5000 bp from TLS)**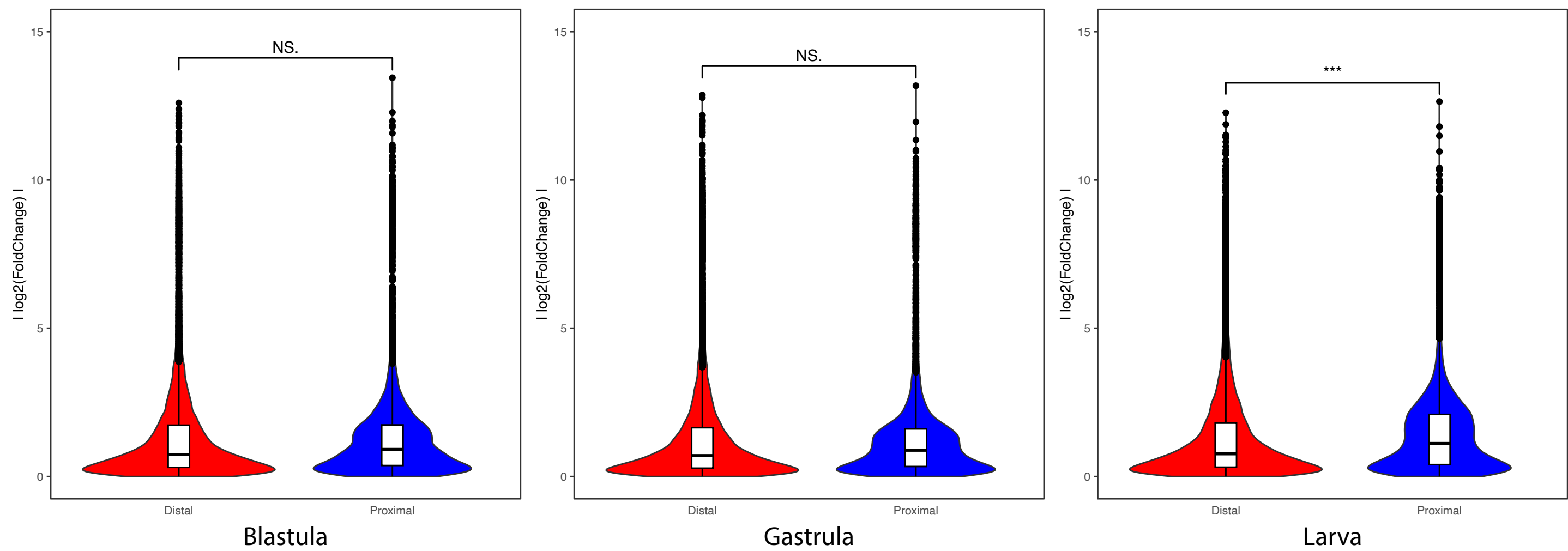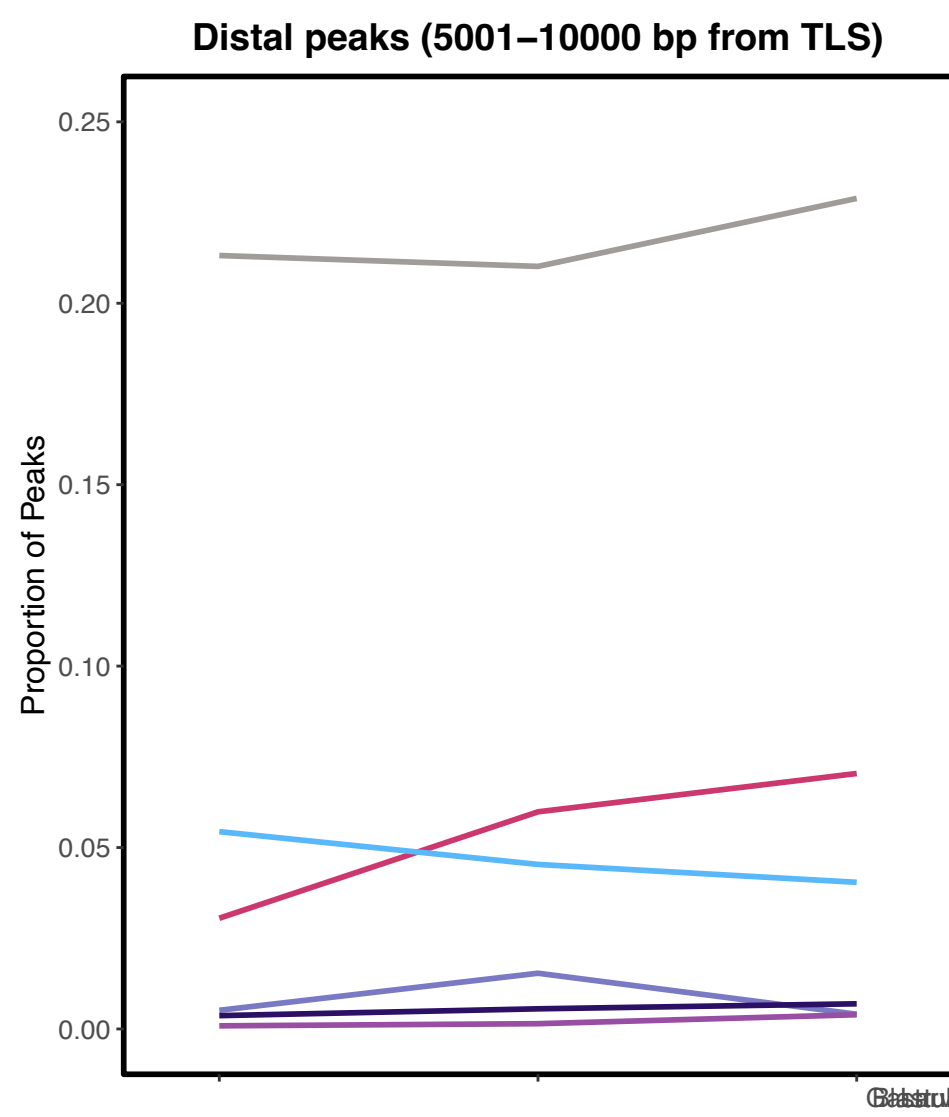**Distal peaks (5001–10000 bp from TLS)**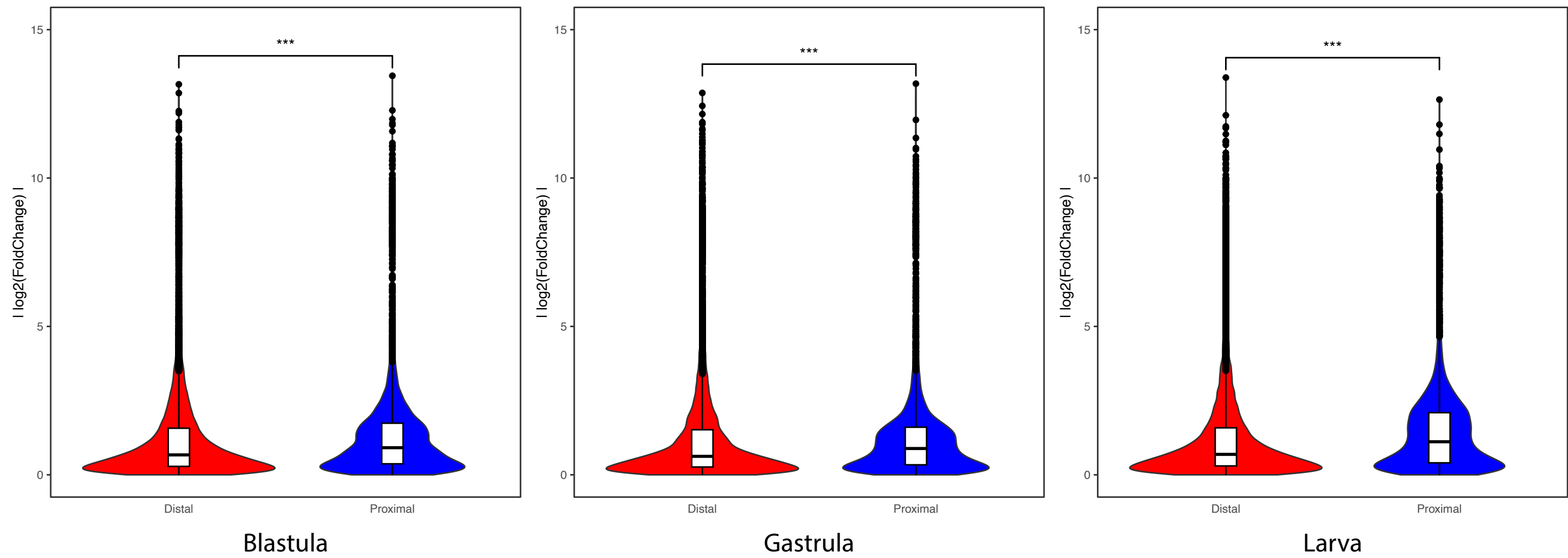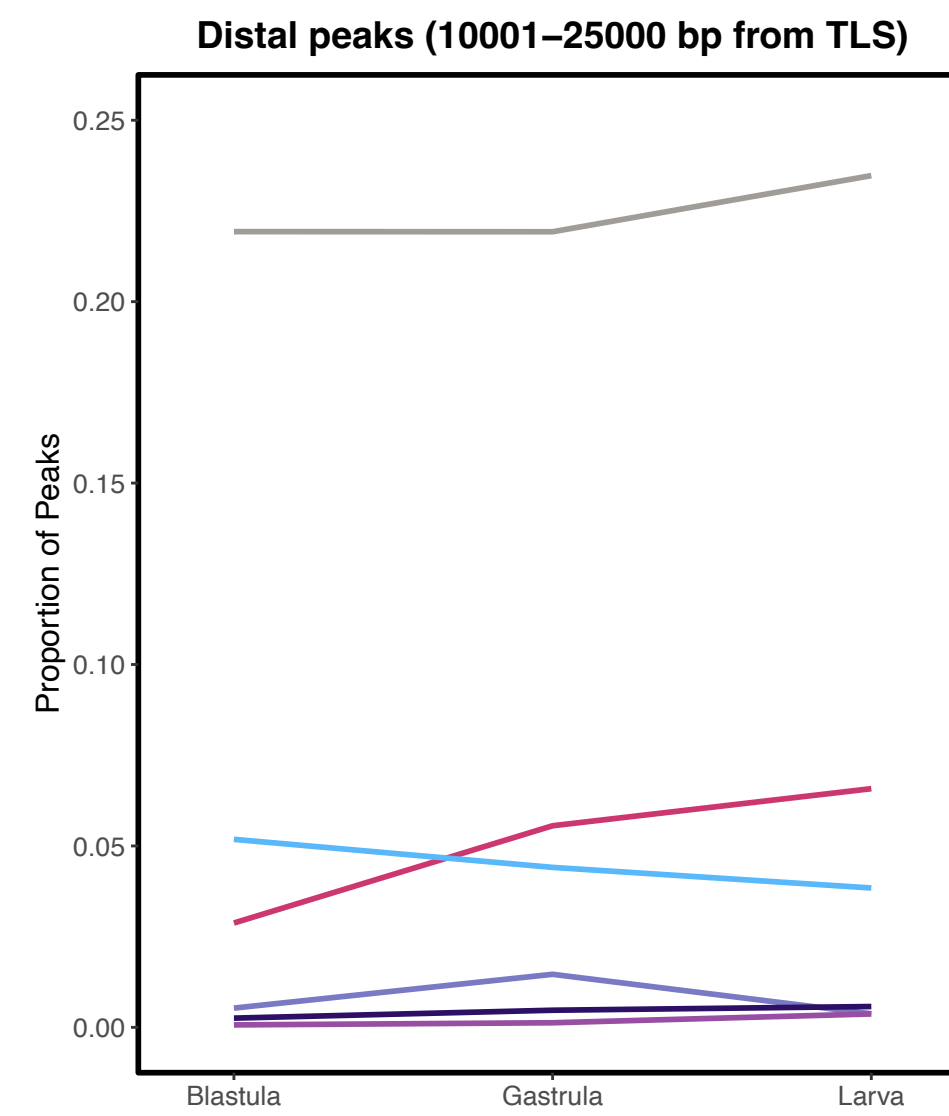**Distal peaks (10001–25000 bp from TLS)**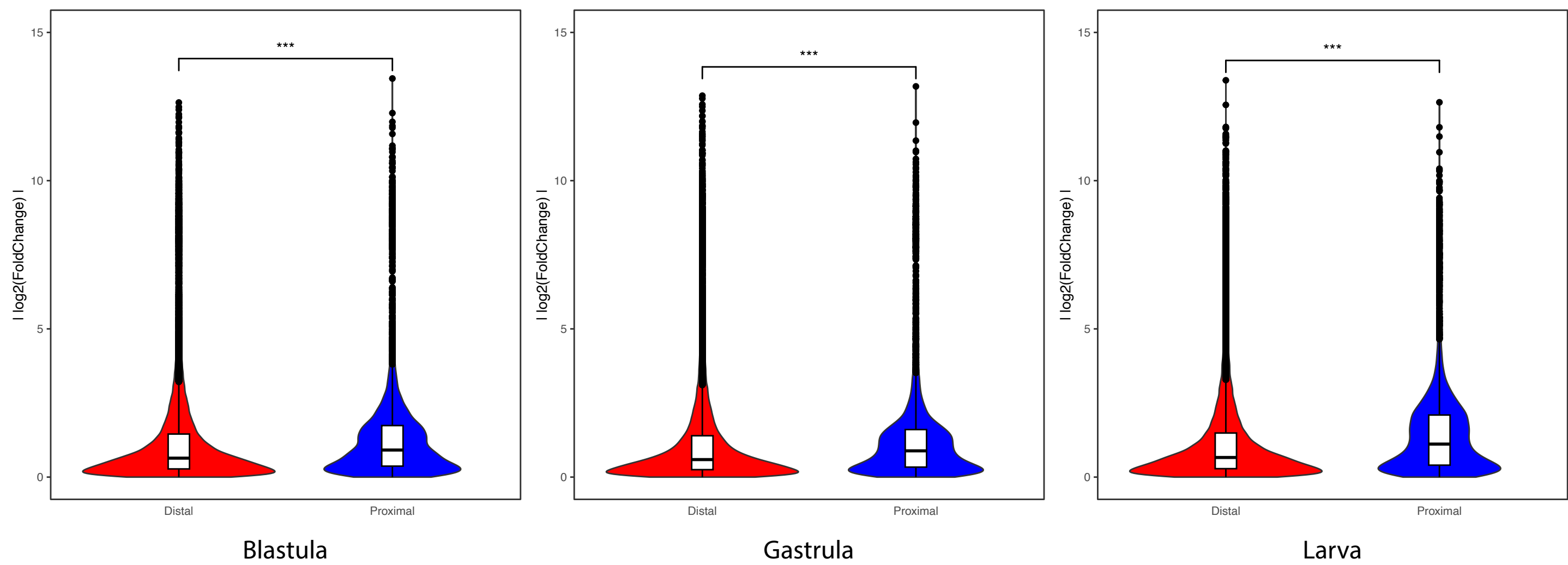

Supplement: msad222_Supplementary_Data [file msad222_supplementary_data.zip › Fig S10_distalbreakdown.pdf]
